# Chronic interferon signaling at baseline is associated with acquired resistance to immunotherapy in metastatic melanoma

**DOI:** 10.1016/j.xcrm.2026.102981

**Published:** 2026-08-07

**Authors:** Donagh Egan, Ben Shum, Rabia Fidan, Walter Kolch, Donal Brennan, Irene Lobon, Samra Turajlic

**Affiliations:** 1 Systems Biology Ireland, University College Dublin, School of Medicine, Belfield, Dublin, Ireland; 2 Cancer Dynamics Laboratory, The Francis Crick Institute, London, UK; 3 Skin and Renal Unit, Royal Marsden NHS Foundation Trust, London, UK; 4 Department of Pathology, Mater Misericordiae University Hospital, Dublin, Ireland; 5 Conway Institute of Biomolecular & Biomedical Research, University College Dublin, Belfield, Dublin, Ireland

**Keywords:** immunotherapy, acquired resistance, durable response, relapse, melanoma, interferon signaling, intratumor heterogeneity

## Abstract

Interferons can trigger robust anti-tumor responses following immune checkpoint blockade (ICB). However, chronic interferon signaling can also reshape tumor cell phenotypes, selecting for immune evasion mechanisms that drive resistance. In a cohort of 108 metastatic melanoma samples collected prior to first-line ICB, immune infiltration and bulk interferon gamma (IFNG) expression correlate with initial but not durable responses. Using a regression-based approach to deconvolve cancer cell signals from bulk tumors, we find that tumors from patients who initially respond but later acquire resistance exhibit heightened cancer cell-dependent IFN signaling at baseline, accompanied by MYC downregulation, dedifferentiation, and impaired major histocompatibility complex (MHC) class II induction. Genetically, acquired resistance also associates with reduced T cell interferon signaling, lower memory T cell activity, high tumor mutational burden, clonal diversification, and elevated subclonal neoantigen burden. These findings reveal baseline transcriptomic and genetic features distinguishing acquired resistance from durable ICB response, identifying candidate targets to prevent relapse and refine stratification.

## Highlights

• A tumor purity-informed model identifies cancer cell programs from bulk RNA-seq
• Chronic cancer cell interferon signaling distinguishes acquired from durable response
• Dedifferentiation impairs MHC class II induction by interferon signaling in resistant tumors
• Durable response associates with low intratumor heterogeneity and effector T cells

## Graphical abstract

**Figure:**
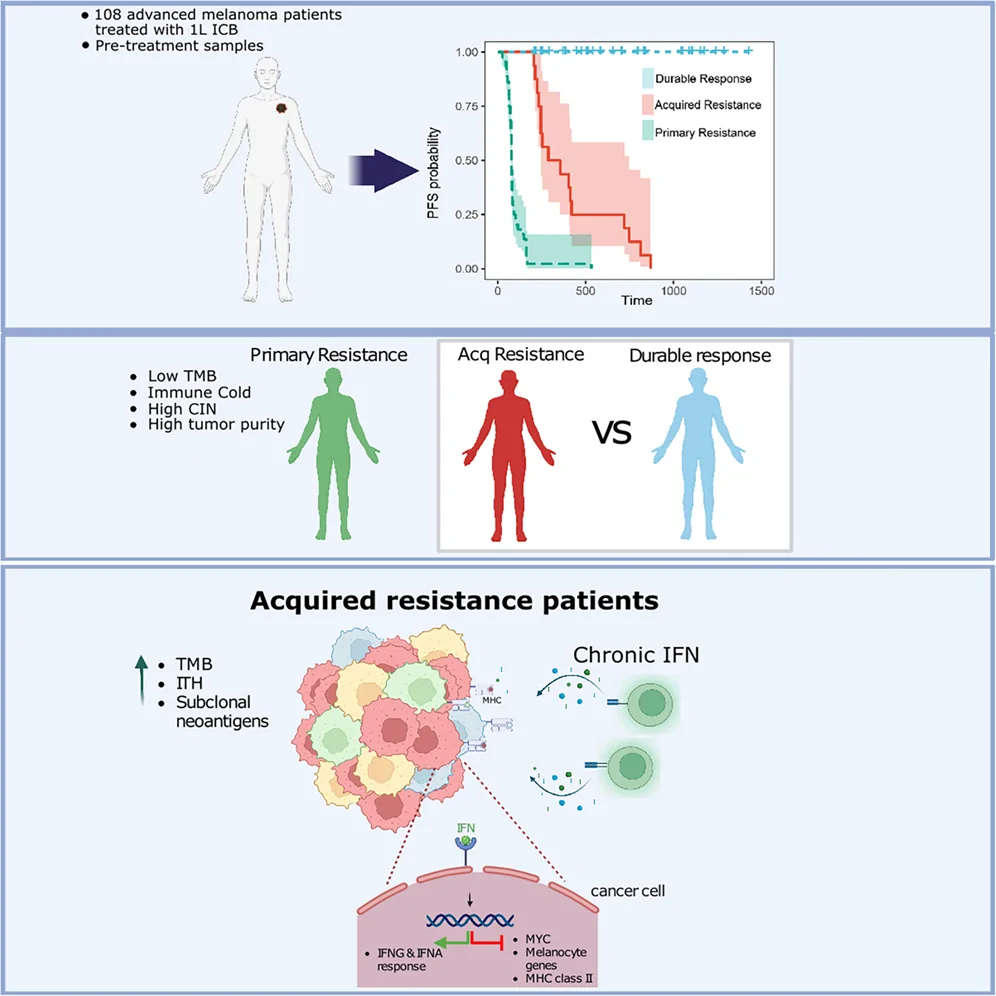

## Teaser

Egan et al. analyze pre-treatment melanoma samples from first-line immune checkpoint blockade patients. Using a tumor purity-informed model to deconvolve cancer cell signals from bulk RNA-seq, they show that chronic interferon signaling drives dedifferentiation and impaired antigen presentation in tumors that later relapse, identifying targets to prevent acquired resistance.

## Introduction

Cancer cells evolve under immune pressure and frequently upregulate immune checkpoint ligands to suppress antitumor immunity. Immune checkpoint blockade (ICB) restores anti-tumor immunity by blocking inhibitory receptors such as programmed cell death protein 1(PD-1) and cytotoxic T-lymphocyte-associated protein 4 (CTLA-4).^1^ Although combined ipilimumab and nivolumab have significantly improved outcomes in metastatic melanoma, 42% of patients experience primary resistance, 24% develop acquired resistance after an initial response, and only 34% achieve durable responses.^2^ Understanding the mechanisms underlying these divergent outcomes is crucial for guiding alternative treatment strategies for transient responders, with the potential to prevent relapse, minimize treatment-related toxicities, and uncover targets.

Several biomarkers have been identified to predict response to ICB, including tumor mutation burden (TMB), tumor clonality, and bulk-derived interferon gamma (IFNG).^3^^,^^4^ However, to date, the primary focus has been predicting response and overall survival, obscuring critical distinctions between primary resistance, acquired resistance, and durable response phenotypes. Furthermore, since ICB was introduced into clinical practice relatively recently, many biomarker studies were conducted in cohorts with mixed treatment histories, including prior exposure to targeted therapies, which can alter immune dynamics and ICB responses.^5^ As first-line ICB is now the standard of care for metastatic melanoma and increasingly used in the neoadjuvant setting, biomarkers derived from treatment-naive samples in patients receiving first-line ICB are urgently needed.

Accumulating evidence suggests that cancer cell-intrinsic interferon signaling is associated with ICB resistance. In a melanoma mouse model, chronic interferon signaling in cancer cells reprograms the epigenome, upregulating interferon-stimulated genes and promoting immune exhaustion.^6^ Resistant lung tumors upregulate interferon signaling within cancer cells,^7^ and interferon treatment of melanoma cells *in vitro* drives dedifferentiation, antigen loss, and reduced T cell activation.^8^ Despite these findings, the relative contributions of immune cell-derived versus cancer cell-intrinsic interferon response across primary resistance, acquired resistance, and durable response patients remain uncharacterized in large, clinically relevant, melanoma patient cohorts.

Although single-cell RNA sequencing offers high-resolution insight into tumor and immune cell states, large treatment-naive first-line ICB cohorts with longitudinal follow-up remain limited. Consequently, bulk RNA-seq remains indispensable for characterizing cancer cell-intrinsic transcriptional profiles at scale in these cohorts. However, tumor microenvironment (TME)-derived signals confound cancer cell-intrinsic expression in bulk RNA-seq. While regression-based approaches can partially extract cell-type-specific profiles from mixed populations,^9^ identifying cancer cell-intrinsic profiles remains challenging due to overlap with stromal transcriptional programs. Recent strategies have leveraged tumor purity estimates from whole-genome sequencing (WGS) or cancer cell expression profiles from scRNA-seq.^10^^,^^11^

Here, we analyzed a cohort of 108 metastatic melanoma samples collected prior to first-line ICB, profiled by bulk RNA-seq and WGS with longitudinal clinical follow-up. This cohort allowed us to identify molecular features associated with primary resistance, acquired resistance, and durable response to ICB. To mitigate the confounding effects of the TME, we implemented a tumor purity-informed linear regression model to identify cancer cell-dependent expression changes associated with response duration. Using this approach, we define baseline transcriptomic and genetic features that distinguish durable response from acquired resistance and reveal chronic cancer cell-driven interferon signaling as a feature implicated in relapse.

## Results

### Cohort description and patient characterization

**Figure 1:**
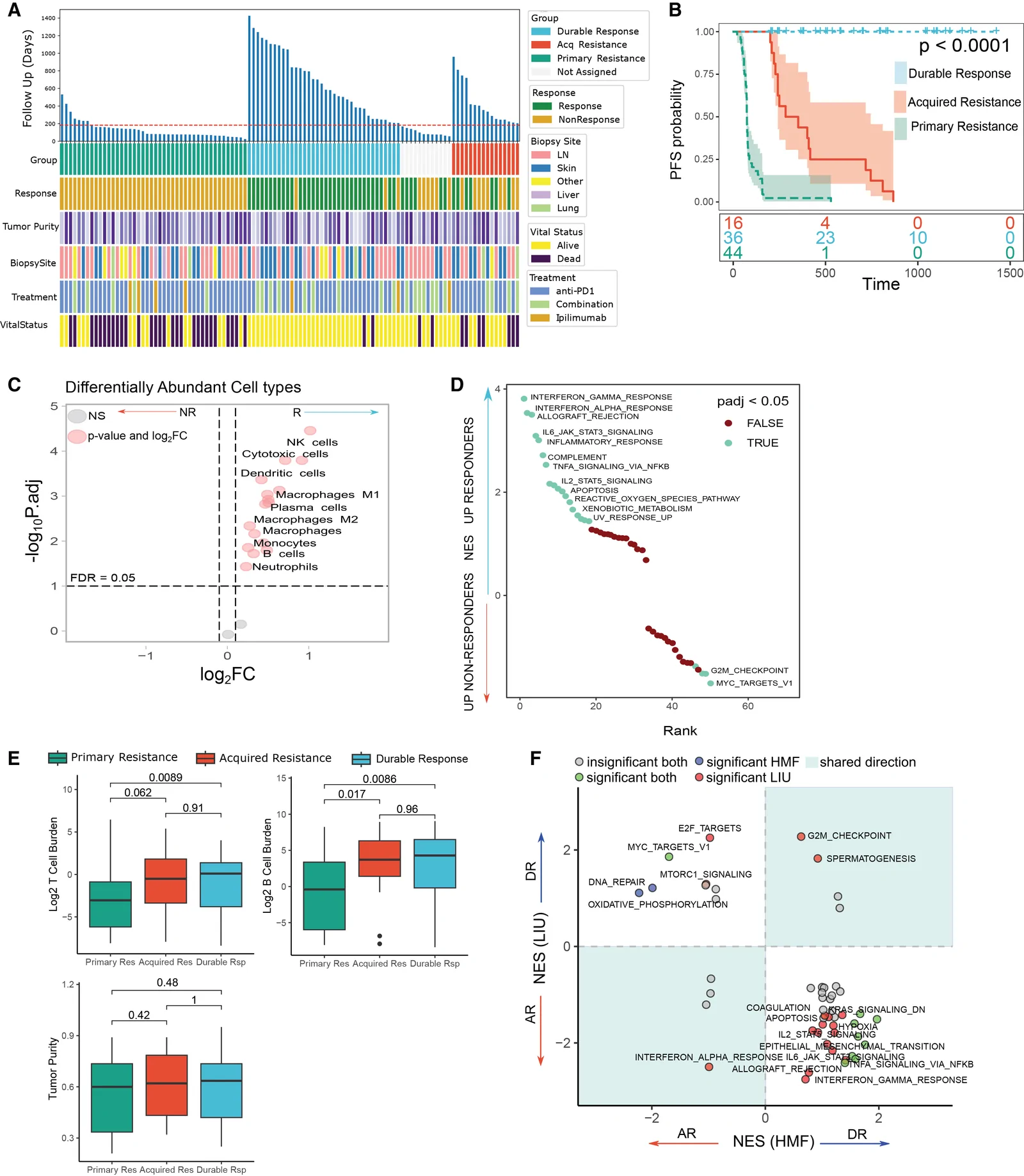
The clinical characteristics of the HMF cohort and transcriptomic features associated with response to first-line ICB (A) Clinical information and response outcomes for patients with bulk RNA-seq data (*n* = 108). Each column represents a patient. Patients are ordered by the duration of response groupings and within each group by duration of response follow-up. The horizontal red line denotes the minimum follow-up required for assignment to a duration of response group (6 months). (B) The progression-free survival (PFS) of patients with bulk RNA-seq data that were assigned to a duration of response group (*n* = 96). Statistical significance was assessed using a log-rank test. (C) Responder and non-responder comparison of ConsensusTME-inferred cell types. A two-sided Wilcoxon rank-sum test with Benjamini-Hochberg (BH) correction was used. (D) Enriched pathways between responders and non-responders, ranked according to normalized enrichment score (NES). Significant pathways (FDR <0.05) are colored in blue. (E) T cell burden (top left), B cell burden (top right), and tumor purity (bottom left) in the duration of response groups. T and B cell burden values represent the relative abundance of clonally rearranged T and B cell transcripts in a tumor. A Wilcoxon rank-sum test was used (two-sided). (F) Enriched pathways between AR and DR groups in the HMF (*x* axis) and Liu datasets (*y* axis). Green dots within the blue squares indicate significant pathways with consistent pathway activity across datasets. The normalized enrichment score (NES) represents pathway activity.

We analyzed a bulk RNA-seq dataset from the Hartwig Medical Foundation (HMF dataset), comprising 108 metastatic melanoma samples collected prior to first-line ICB therapy. Samples were derived from lymph nodes (*n* = 50), skin/subcutaneous tissue (*n* = 39), lung (*n* = 5), liver (*n* = 4), and others (*n* = 10) involved by melanoma (Figure 1A). Principal component analysis (PCA) of gene expression profiles revealed no clustering by sample site, suggesting it is not a major source of variation in the data (Figure S1A). Furthermore, the distribution of sample sites was consistent between the clinical groups compared in downstream analyses (chi-squared test, *p* > 0.1). Patients received anti-PD1 (70%), anti-CTLA4 (7%), or a combination of both (23%). Based on RECIST criteria, 43% of patients were responders, defined as partial (PR) or complete response (CR), and 57% were non-responders, defined as stable (SD) or progressive disease (PD) (Figure 1A). After a median follow-up of 17 months, the progression-free survival (PFS) was 5.5 months, and the median overall survival (OS) was not reached. Additional clinical characteristics of this cohort are detailed in Table S1. All datasets used in this study and their respective purposes are detailed in Figure S1B.

To identify determinants of response durability, a major unmet need, patients were categorized into three groups: primary resistance (*n* = 44), acquired resistance (AR, *n* = 16), and durable response (DR, *n* = 36). Patients in the DR and AR groups were required to have response or maintain SD for at least 6 months; AR patients were those who progressed after 6 months and while on treatment, whereas DR patients remained progression-free during follow-up (Figure 1B). Patients classified as having primary resistance demonstrated disease progression or SD of less than 6 months as best response. Patients with insufficient follow-up (less than 6 months, *n* = 12) were excluded from duration-of-response analyses (Figure 1A).

### Tumor-immune composition correlates with response to first-line ICB

We first compared the TME between responders and non-responders by deconvolving the abundance of different cell types.^12^ Consistent with previous studies, responders exhibited higher immune cell infiltration compared to non-responders, particularly natural killer (NK) cells and cytotoxic T cells (false discovery rate [FDR] <0.05) (Figure 1C).^13^^,^^14^ Tumor purity was correspondingly numerically higher in non-responders compared to responders (*p* = 0.13) (Figure S1C). No differences were observed between the non-immune components of the stroma, namely endothelial cells and fibroblasts (Figure 1C). As tumor purity and immune cell infiltration were consistent across sample sites (Figures S1D; Table S2), it is unlikely that variation between sample sites confounded the observed associations.

Next, we quantified T and B cell infiltration with an orthogonal approach that assembles rearranged T cell receptor (TCR) and immunoglobulin (Ig) transcriptomic reads.^15^ High TCR and Ig read counts were correlated with response, but only TCR reads reached statistical significance (*p* = 0.046 and *p* = 0.067, respectively) (Figure S1C; Table S3). Gene set enrichment analyses (GSEA) identified immune-related pathways including IFNG response as highly upregulated in responders (Figure 1D; Figure S1E; Table S4). Similarly, an IFNG-related signature identified by Ayers et al.,^4^ which has been validated in clinical trials for metastatic melanoma,^16^ demonstrated significant upregulation in responders (*p* < 0.01) (Figure S1F). In non-responders, MYC targets and the G2M checkpoint were the most significantly upregulated pathways (Figure 1D) but were also among the pathways most positively correlated with tumor purity (Figure S1G). Together, these findings demonstrate that the differences in bulk-level pathway activity between responders and non-responders are explained by higher immune infiltration and lower tumor purity in responders.

### Immune activation predicts initial response but not long-term durability

Extensive research efforts have identified biomarkers of ICB response; however, the features underlying durable responses—the ultimate goal of therapy—remain unclear. This gap reflects the recent introduction of ICB in clinical practice, limitations of available datasets with sufficient follow-up, and cohort heterogeneity, as discussed above. To address this, we compare the DR and AR groups in the HMF cohort.

Consistent with clinical trial outcomes,^2^ a numerically higher proportion of patients in the DR group (29%) received combination anti-PD1 and anti-CTLA4 compared to the AR (17%) and primary resistance (18%) groups (chi-squared test, *p* = 0.316) (Figure S1H).

The abundance of all cell types within the TME, as determined by cellular deconvolution,^12^ was comparable between DR and AR groups (Figure S1I). Similarly, there were no significant differences in rearranged TCR and Ig transcriptomic reads between groups (Figure 1E). In contrast, tumors from patients with primary resistance exhibited significantly lower levels of TCR and Ig reads, suggesting that immune infiltration estimates derived from bulk RNA-seq data may predict initial response rather than its durability (Figure 1E). Consistent with this interpretation, the previously established IFNG-related signature^4^ failed to differentiate DR and AR patients in the HMF cohort (Figure S1J), indicating either a reduced capacity to predict durable response or that at the bulk level, the signature primarily reflects overall immune cell infiltration, which is comparable between DR and AR patients (Figure 1E; Figure S1I).

GSEA identified KRAS signaling, epithelial to mesenchymal transition, and inflammatory response as being upregulated in DR compared to AR patients (Figures 1F and X-axis). To validate these findings, we analyzed an independent dataset published by Liu et al.,^17^ selecting treatment-naive metastatic melanoma samples to ensure consistency with the HMF cohort. Patients were classified based on PFS as having primary resistance (*n* = 24), AR (*n* = 12), and DR (*n* = 21) following first-line anti-PD1 (Figure S1K; Table S5; detailed in methods). We did not recapitulate any pathway associations with durable response in the HMF dataset in this cohort (Figure 1F). This discrepancy is potentially explained by differences in tumor purity and TME composition between datasets. In the Liu dataset—but not in the HMF dataset—AR patients demonstrated lower tumor purity (*p* = 0.013), higher immune cell infiltration, and increased expression of the previously established IFNG-related signature^4^ compared to DR patients (Figures S1L–S1N). Notably, many pathways that appeared enriched in the Liu dataset lost statistical significance after controlling for tumor purity (Figure S2A). Furthermore, the established IFNG-related signature^4^ was not associated with initial response in the Liu dataset, as elevated expression in AR patients was offset by lower expression in DR patients (*p* = 0.97) (Figures S2B and S1M).

These findings highlight a well-established characteristic of bulk RNA-seq data that warrants consideration when validating gene signatures for clinical use: the primary source of inter-patient variation often reflects differences in tumor purity and TME composition.^18^^,^^19^ As a result, predictive signatures confounded by tumor purity differences may fail to generalize across independent cohorts or routine clinical samples, where purity varies widely.^18^ We hypothesized that biological signals driving durable response would remain consistent across datasets if these confounding effects were mitigated. Therefore, we developed a linear regression model based on established deconvolution algorithms^9^^,^^10^^,^^11^^,^^20^ to identify cancer cell-dependent transcriptional programs associated with durable response that are conserved across datasets. By focusing on cancer cell-dependent signals, our approach reduces the influence of sampling artifacts and enables the identification of genuine resistance mechanisms that represent potential therapeutic targets.

### The cancer cell-dependent model resolves cancer cell-intrinsic transcriptional signals

**Figure 2:**
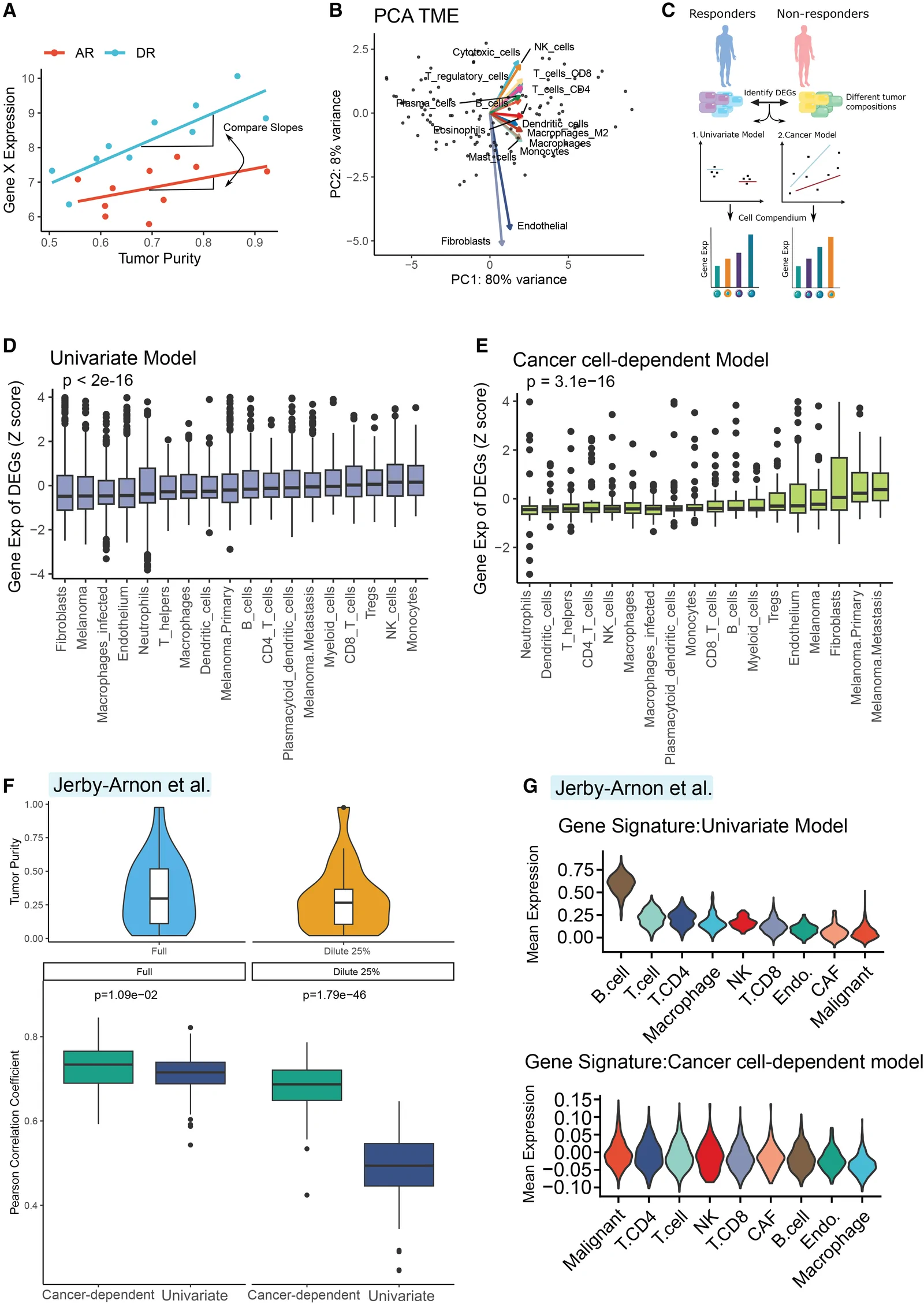
Description and validation of the cancer cell-dependent expression model (A) An illustrative example of the linear regression model used. The slopes of the relationship between tumor purity and gene expression are compared between DR and AR patients. (B) Principal component analysis of Consensus TME-inferred cell type abundances. The feature loadings for PC1 and PC2 are overlaid as arrows and colored according to cell type. (C) The approach used to compare a standard univariate model with the cancer cell-dependent model. DEGs are identified between responders and non-responders using both models and mapped to a compendium of sorted cell types. (D and E) Expression of DEGs (FDR <0.1) identified by comparing responders and non-responders within a compendium of bulk RNA-seq profiles from sorted cell types. DEGs were derived using both the standard univariate model and the cancer cell-dependent model. A Kruskal–Wallis test was used. (F) Correlation of fold-change values estimated by the univariate and cancer cell-dependent models with the ground-truth fold-change values in malignant cells. This comparison was conducted using the full dataset of malignant cells and with a 25% reduction in malignant cell count (dilute 25%). A Wilcoxon rank-sum test was used (two-sided). (G) The average expression of the top 100 genes identified by the standard univariate and cancer cell-dependent models using the full Jerby-Arnon dataset.^21^

We estimated the proportion of cancer cells in each sample (tumor purity) using WGS matched with the bulk RNA-seq data. To identify conserved cancer cell-dependent expression programs associated with response duration, we implemented a linear regression model to identify genes whose relationship with tumor purity differed according to response group (AR or DR; Figure 2A, illustrative example). As a gene’s total expression also reflects its stromal compartment contribution, PCA was performed on the deconvolved TME to identify primary sources of variation, which were incorporated as covariates in the model (Figure 2B, see methods).

To validate this approach, we compared differentially expressed genes (DEGs) identified between responders and non-responders using two models: our cancer cell-dependent model and a standard univariate model that does not account for cell type composition in the TME (illustrated in Figure 2C). DEGs identified with the standard univariate model were upregulated in sorted immune cell populations from a cell type compendium of 4,212 bulk RNA-seq profiles,^22^ reflecting differences in TME composition (*p* < 0.01) (Figure 2D). For example, the DEGs were strongly upregulated in NK cells—the most differentially abundant cell type between responders and non-responders (Figure 1C). Conversely, the DEGs identified with the cancer cell-dependent model were upregulated in metastatic melanoma cells, consistent with their metastatic origin, and exhibited less confounding from immune cells (*p* < 0.01) (Figure 2E).

We further validated this approach using a melanoma scRNA-seq dataset published by Jerby-Arnon et al. (detailed in methods and Figure S2C).^21^ Tumor samples were first clustered according to the proportion stromal cell types (immune cells, endothelial cells, and cancer-associated fibroblasts [CAFs]), identifying two distinct TME groups (Figure S2D). This classification ensures that TME composition contributes to intergroup variation. DEGs were then identified between the malignant cells of both groups, establishing a ground truth for cancer cell-intrinsic differences. Next, the stromal and malignant cells were mixed *in silico* to simulate tumor bulk RNA-seq samples, and DEGs were identified between both groups using either the standard univariate model or the cancer cell-dependent model. Fold change estimates from the cancer cell-dependent model showed significantly stronger correlation with the ground truth compared to the univariate model, particularly when malignant cells in the simulated samples were artificially diluted by 25% (*p* < 0.01) (Figure 2F). This performance gap reflects the cellular origin of the identified DEGs: the standard univariate model identified genes upregulated in B cells—a population that differed in abundance between groups (Figure S2D)—whereas the cancer cell-dependent model identified genes upregulated in malignant cells (Figure 2G). This demonstrates that the cancer cell-dependent model prioritizes cancer cell-intrinsic transcriptional signals by accounting for differences in cell-type composition that confound standard bulk RNA-seq analyses.

### Cancer cell-dependent interferon signaling is associated with acquired resistance to ICB

**Figure 3:**
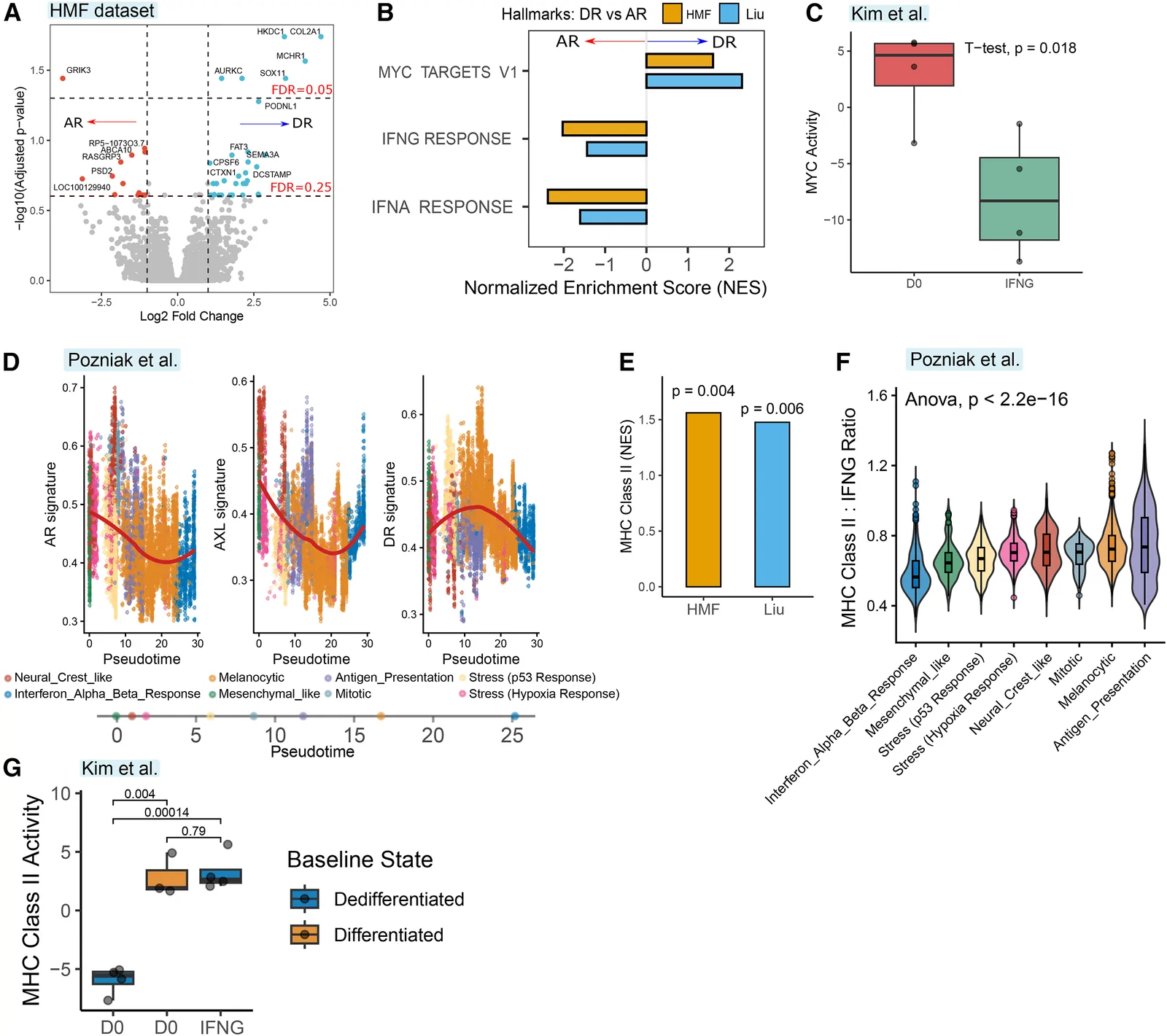
The cancer cell-dependent transcriptional programs associated with duration of response (A) The DEGs between DR and AR patients using the cancer cell-dependent model in the HMF dataset. B) Enriched pathways driven by the cancer cells of DR or AR patients that are conserved between the HMF and Liu et al. datasets. An FDR threshold of 0.05 was applied. (C) The pathway activity of MYC targets in differentiated human melanoma cell lines before and after IFNG treatment for 2 to 5 weeks^31^ A two-sided *t* test was used based on the Shapiro-Wilk test for normality. (D) The AR, AXL, and DR signatures scored in melanoma cells ordered by pseudotime.^32^ The pseudotime scores for each cell phenotype are represented by a dot on the one dimensional timeline. (E) MHC class II pathway activity associated with cancer cells in DR compared to AR patients. The normalized enrichment score (NES) was determined using GSEA on the Reactome database. (F) The ratio of MHC class II pathway activity to IFNG response in melanoma cells from Pozniak et al.^32^ (G) MHC class II pathway activity in baseline differentiated human melanoma cell lines and in baseline dedifferentiated cell lines before and after 2–5 weeks of IFNG treatment.

Having validated the model to identify cancer cell-dependent signals in bulk samples, we applied it to compare DR and AR patients and identify DEGs associated with durable responses. In the HMF dataset, *MCHR1*, *SOX11* and *COL2A1* were strongly downregulated in AR patients (FDR <0.05) (Figure 3A; Table S6). *MCHR1* is a melanocyte autoantigen implicated in vitiligo,^23^ and germline mutations in *SOX11* cause Coffin-Siris syndrome,^24^ a condition linked to vitiligo in some patients.^25^^,^^26^^,^^27^ *COL2A1* upregulates TYR, the rate-limiting enzyme in melanin synthesis,^28^ and can itself act as an autoantigen when post-translationally modified, as observed in rheumatoid arthritis.^29^ Consistent with these findings, both COL2A1 and TYR were downregulated in AR patients in the Liu dataset (nominal *p* value <0.05) (Table S7). Collectively, these results suggest that cancer cells in AR patients exhibit reduced melanocyte differentiation and diminished expression of potentially immunogenic proteins, consistent with a dedifferentiated state previously linked to immunotherapy resistance.^30^

To explore this further, we mapped cancer cell-dependent DEGs to the Hallmarks database using GSEA. In both the HMF and Liu datasets, IFNG and interferon alpha (IFNA) responses were upregulated in AR patients, while MYC targets were downregulated (FDR <0.05) (Figure 3B) (Tables S8 and S9). Inflammatory signaling can induce melanoma dedifferentiation,^30^ whereas MYC promotes terminal differentiation by driving stem cells toward transit-amplifying cells.^33^^,^^34^^,^^35^^,^^36^^,^^37^ We therefore hypothesized that elevated IFNG within the TME of AR patients triggers a cancer cell IFNG response that promotes dedifferentiation and downregulation of MYC activity. We confirmed this in a dataset published by Kim et al.,^31^ in which differentiated human melanoma cell lines (M262, M308, M399, and 3998MEL) treated with IFNG for 2–5 weeks exhibited significantly reduced MYC activity (*p* = 0.018; Figure 3C).

Melanoma cells transition through four differentiation states—undifferentiated, neural crest-like, transitory, and melanocytic—each exhibiting distinct sensitivity to therapy.^38^ To determine the cancer cell differentiation state associated with the DR and AR groups, we created gene signatures from cancer cell-dependent genes significantly upregulated in each group (FDR <0.1; Table S10). These signatures were then scored in a pre-treatment scRNA-seq dataset from Pozniak et al.^32^ Using the original annotations, melanoma cells were ordered along a pseudotime trajectory representing progressive differentiation (Figures S2E and S2F). The AR signature was highest in dedifferentiated mesenchymal stem and neural crest cells, declined toward melanocytic, and increased again in the interferon alpha-beta response phenotype (Figure 3D). Notably, an established AXL-high melanoma dedifferentiation signature followed a similar trajectory to the AR signature (Figure 3D).^39^ Conversely, the DR signature was lowest in dedifferentiated cells, peaked at melanocytic, and declined in the IFNA–IFNB response phenotype (Figure 3D). These findings indicate that melanoma cells from DR and AR patients exhibit a differentiated and dedifferentiated phenotype, respectively.

IFNG signaling in cancer cells can promote antigen presentation via the induction of MHC class I and class II expression but may also promote immune evasion through upregulation of *PDL1*, which suppresses the anti-tumor immune response.^40^^,^^41^ In both the HMF and Liu datasets, AR patients exhibited no difference in MHC class I activity, a significant reduction in MHC class II activity (*p* < 0.01; Figure 3E), and a trend toward increased *PD-L1* expression compared to DR patients (*p* = 0.15 and *p* = 0.13 in the HMF and Liu dataset, respectively; meta-analysis *p* = 0.096; Figure S2G). Given the reduced MHC class II activity in AR tumors despite elevated IFNG signaling, we hypothesized that melanoma differentiation state may account for this discrepancy. Indeed, differentiated melanocytic cells from Pozniak et al.^32^ exhibited high MHC class II activity relative to IFNG signaling, whereas the interferon alpha-beta response and mesenchymal phenotypes displayed the lowest MHC class II activity relative to IFNG signaling (Figure 3F). Similarly, in the Kim et al. dataset,^31^ melanoma cell lines with a differentiated phenotype at baseline exhibited significantly higher MHC class II activity than dedifferentiated lines (M257A2, M370, M381, and M410; *p* < 0.01) (Figure 3G). Following 2 to 5 weeks of IFNG treatment, MHC class II activity in dedifferentiated cell lines increased to levels comparable to those of the differentiated lines (Figure 3G). These findings suggest that dedifferentiated melanoma cells, such as those associated with AR patients, display reduced MHC class II activity relative to interferon signaling, linking cellular differentiation state to antigen presentation capacity and, ultimately, response duration.

### Interferon signaling associated with cancer cells and T cells exerts opposing effects in DR and AR patients

**Figure 4:**
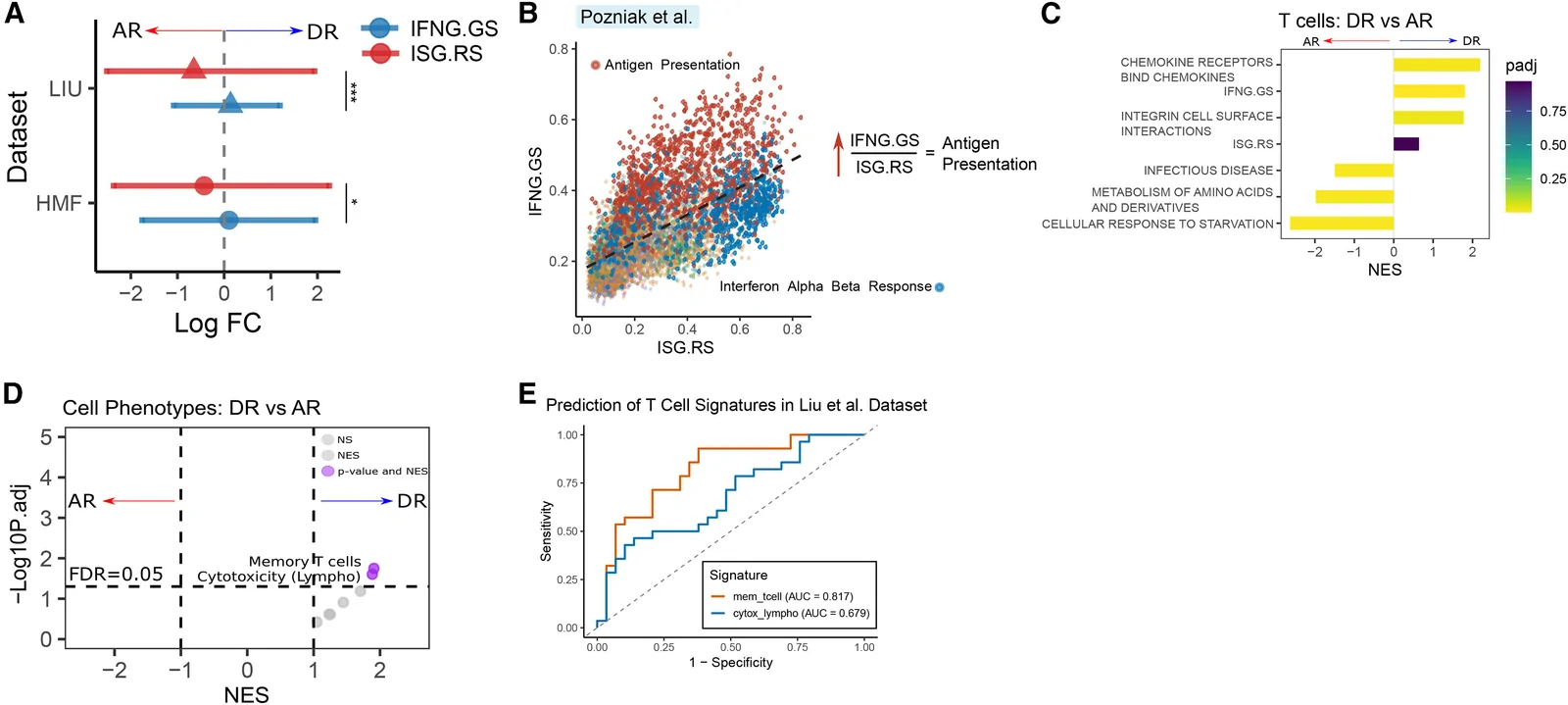
Differential expression of cell type-enriched interferon signatures in DR and AR patients (A) The fold change values, identified with the cancer cell-dependent model, for interferon-related genes that were previously identified as enriched in cancer cells (ISG.RS) or immune cells (IFNG.GS) (∗*p* < 0.05, ∗∗∗*p* < 0.001).^42^ A Wilcoxon rank-sum test was used. (B) The relationship between IFNG.GS and ISG.RS expression in melanoma cells from Pozniak et al., colored by cell phenotype.^32^ (C and D) Enriched pathways and phenotypes driven by T cells in DR versus AR patients in the HMF dataset, respectively. The cell phenotype signatures were accessed from Sade Feldman et al.^13^ (E) ROC curves evaluating the performance of HMF-derived T-cell-dependent genes in predicting cytotoxic and memory T cell signatures in the Liu et al. cohort. High versus low cell-type scores were defined based on median values.

Minn and colleagues described opposing effects of interferon signaling in cancer versus immune cells, identifying distinct gene subsets within the Hallmark IFNG response signature: a cancer cell-enriched interferon signature (ISG.RS), associated with ICB resistance, and an immune-cell-enriched interferon signature (IFNG.GS) associated with respone.^42^ In both the Liu and HMF datasets, fold change values for ISG.RS genes, derived from the cancer cell-dependent model, were elevated in AR relative to DR patients and were significantly upregulated compared to IFNG.GS genes (*p* < 0.05) (Figure 4A). These results indicate that ISG.RS genes are the predominant contributors to the increased interferon signaling associated with cancer cells in AR patients.

Given the differential signaling of ISG.RS and IFNG.GS in AR patients, we analyzed these signatures in the scRNA-seq dataset from Pozniak et al.^32^ As expected for genes within the same Hallmark signature, the two signatures were correlated. However, the relative expression of the IFNG.GS to the ISG.RS was notably higher in melanoma cells with an antigen presentation phenotype compared to those with an interferon alpha-beta response phenotype (Figure 4B). Therefore, the elevated expression of the ISG.RS relative to the IFNG.GS in AR patients is consistent with an interferon alpha-beta response phenotype rather than an antigen presentation phenotype.

To characterize the T cell compartment in DR and AR patients, we adapted the cancer cell-dependent model to identify differentially active pathways driven by T cells (HMF dataset; detailed in Methods). Briefly, T cell abundance, inferred using MiXCR, was used to identify genes whose correlation with T cell levels differed according to response groups (AR or DR). Compared to AR, the DR patients upregulated the IFNG.GS, integrin interaction pathways, and chemokine receptor binding, whereas the ISG.RS did not reach statistical significance (Figure 4C). In contrast, AR patients upregulated amino acid metabolism and cellular responses to starvation, indicative of metabolic rewiring (Table S11). Mapping these results to T cell phenotype signatures previously defined in ICB-treated melanoma patients^13^ revealed significant upregulation of cytotoxic and memory T cells in DR versus AR patients (Figure 4D). Consistent with these findings, the T cell-dependent genes upregulated in DR patients independently predicted the cytotoxic and memory T cell signature scores in the Liu et al. dataset (area under the curve [AUC] = 0.68 and 0.81, respectively; Figure 4E). Together, these findings are consistent with interferon signaling exerting cell type-specific effects in melanoma. In DR patients, cancer cells exhibit reduced ISG.RS activity, whereas T cells display elevated IFNG.GS signaling, response to chemokines, and effector phenotypes. In contrast, AR tumors are characterized by heightened interferon signaling in cancer cells accompanied by metabolic rewiring in T cells. These opposing effects are obscured in bulk analyses, highlighting the need for cell type-resolved approaches to identify candidate biomarkers of durable response for evaluation in prospective studies.

### Cancer cells in primary resistance patients are associated with interferon signaling and a mesenchymal phenotype

**Figure 5:**
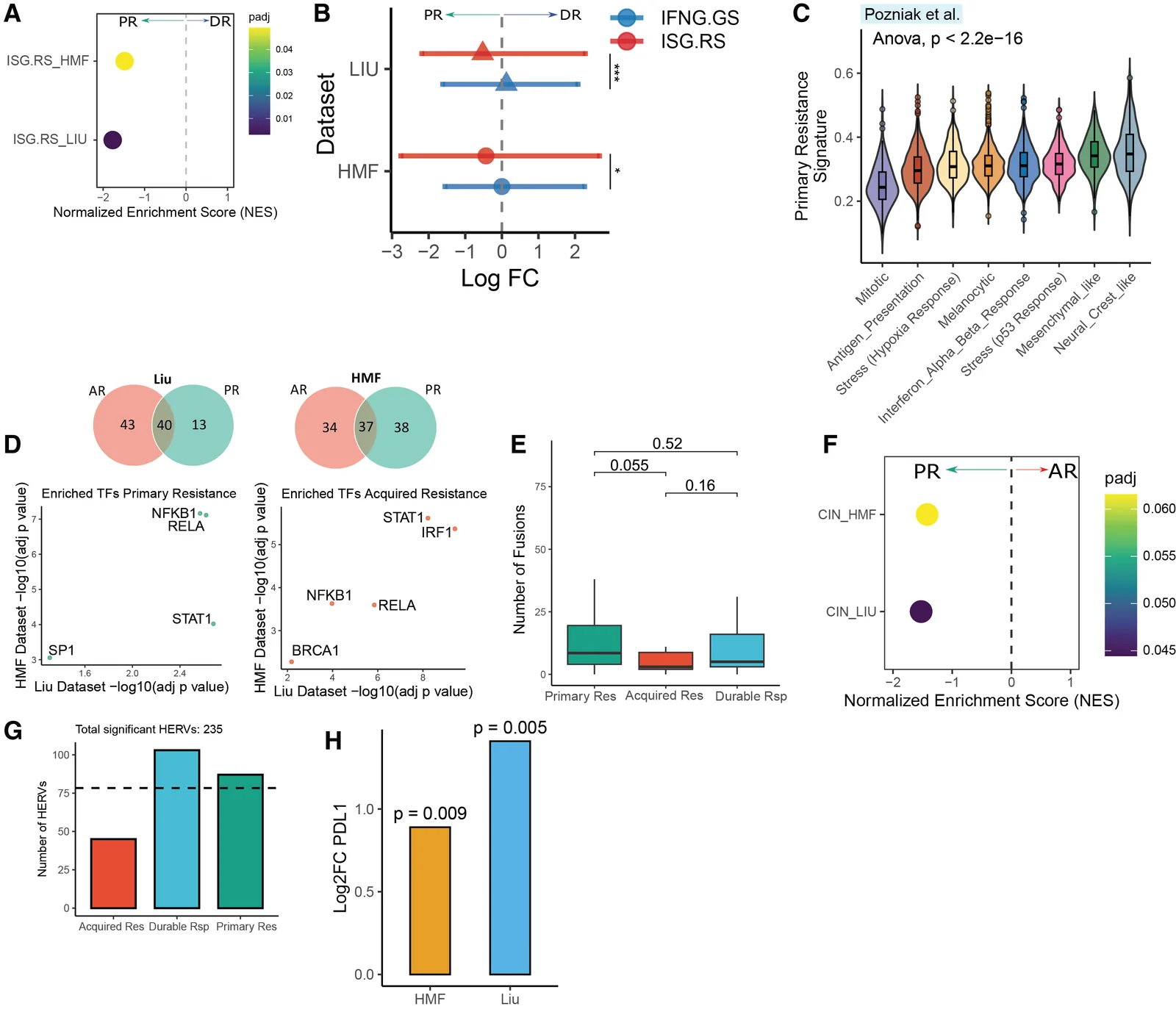
Cancer cell-dependent transcriptional programs associated with primary resistance (A) The cancer cell-dependent pathway activity (NES) of the ISG.RS in DR compared to primary resistance patients. (B) The fold change values, identified with the cancer cell-dependent model, for interferon-related genes that were previously identified^42^ as enriched in cancer cells (ISG.RS) or immune cells (IFNG.GS) (∗*p* < 0.05, ∗∗∗*p* < 0.001). A Wilcoxon rank-sum test was used. (C) Expression of the primary resistance signature across the melanoma phenotypes identified in Pozniak et al.^32^ The primary resistance signature was derived by comparing DR and primary resistance patients in the Liu and HMF datasets (see Methods for details). (D) Top panel, the overlap of IFNG leading-edge genes between AR and primary resistance patients in the Liu and HMF datasets. Bottom panel, the transcription factor enrichment significance level [-log10(adj *p* value)] for the leading genes unique to AR or primary resistance patients across both datasets. (E) Gene fusion estimates for the duration of response groups in the HMF dataset. Statistical significance was calculated using a Wilcoxon rank-sum test (two-sided). (F) The pathway activity (NES) of a published signature for characterizing CIN-high versus CIN-low tumors in primary resistance versus AR patients, derived using the cancer cell-dependent model.^43^ (G) Distribution of HERV loci that vary significantly across duration of response groups, categorized by the group with highest expression. The dashed line represents the expected number of upregulated HERVs per group under the null hypothesis of equal distribution. (H) The difference in *PDL1* expression between AR and primary resistance patients (reference group) using the cancer cell-dependent model. Nominal p, uncorrected (*a priori* candidate gene).

To reconcile our findings across all treatment outcomes, we analyzed the IFNG.GS and the ISG.RS in primary resistance versus DR patients using the cancer cell-dependent model. Similar to AR patients, the ISG.RS was significantly upregulated in primary resistance compared to DR patients in both the HMF and Liu datasets, whereas the IFNG.GS showed no significant difference (Figure 5A). Similarly, fold change values for ISG.RS genes were elevated in primary resistance relative to DR patients and were significantly upregulated compared to IFNG.GS genes (Figure 5B). These results suggest that despite having distinct immune infiltration levels at the time of sampling, the ISG.RS is the dominant interferon signal associated with cancer cells in both AR and primary resistance patients compared to DR patients.

Next, we defined a primary resistance signature as cancer cell-dependent genes upregulated in primary resistance versus DR patients (FDR <0.1; Table S10). In the scRNA-seq dataset from Pozniak et al.,^32^ the primary resistance signature did not correlate with pseudotime; however, similar to the AR signature, it was highly expressed in dedifferentiated mesenchymal and neural crest cell phenotypes (Figure 5C), consistent with the original report that the mesenchymal phenotype is associated with primary resistance to ICB.^32^

To determine whether cancer cells drive similar IFNG responses in primary resistance and AR patients, we compared the transcription regulatory networks underlying IFNG response in each group. While several enriched transcription factors (TFs) were shared, the order was different and consistent across datasets: STAT1 and IRF1 were the top TFs in AR, while RELA and NFKB1 were the most prominent TFs in primary resistance patients (Figure 5D). This result suggests a stronger shift toward NFKB signaling in primary resistance.

Despite an immune-cold phenotype, primary resistance patients exhibited comparable cancer cell-dependent IFNA and IFNG pathway activity to AR patients (data not shown). Combined with the regulatory shift toward NFKB, this suggests that cytosolic nucleic acid sensing may be driving a cell-intrinsic interferon response in primary resistance patients. To explore potential sources, we first quantified gene fusions as a measure for chromosomal instability (CIN), a known activator of the cGAS-STING-NF-κB pathway and interferon response.^43^ Gene fusion burden demonstrated a near-significant increase in primary resistance versus AR patients (*p* = 0.055) (Figure 5E). Furthermore, a previously defined CIN-high gene signature derived from breast cancer was upregulated in primary resistance relative to AR patients in the cancer cell-dependent model (*p* = 0.06 and *p* = 0.042 in the HMF and Liu dataset, respectively; meta-analysis *p* = 0.017) (Figure 5F).^43^

Next, we quantified the global expression of human endogenous retroviruses (HERVs), which have been implicated in sustaining cell-intrinsic interferon signaling in ICB-resistant mouse melanoma.^44^ We identified 235 HERV loci that were differentially expressed across the duration of response groups (Figure S2H) (FDR <0.05, likelihood ratio test). These loci were predominantly upregulated in DR (*n* = 103) and primary resistance (*n* = 87) patients compared to AR patients (*n* = 45; binomial test, *p* < 0.05) (Figure 5G).

Together, these findings suggest that in primary resistance patients, HERV expression and CIN provide a potential substrate to activate a cell-intrinsic interferon response. Notably, downstream DNA and RNA sensors (STING, MDA5, RIG-I) showed comparable expression across patient groups, indicating that activation may occur post-transcriptionally. The association between HERV expression and durable clinical benefit to ICB in preclinical melanoma models^45^^,^^46^ may explain the higher HERV expression in DR versus AR patients.

Finally, *PDL1* expression associated with cancer cells was significantly upregulated in AR patients compared to primary resistance patients, as previously observed in lung cancer patients (*p* < 0.05) (Figure 5H).^7^ Combined with greater immune infiltration, this potentially explains the initial treatment response observed in AR patients.

### Interferon signaling in the neoadjuvant setting

**Figure 6:**
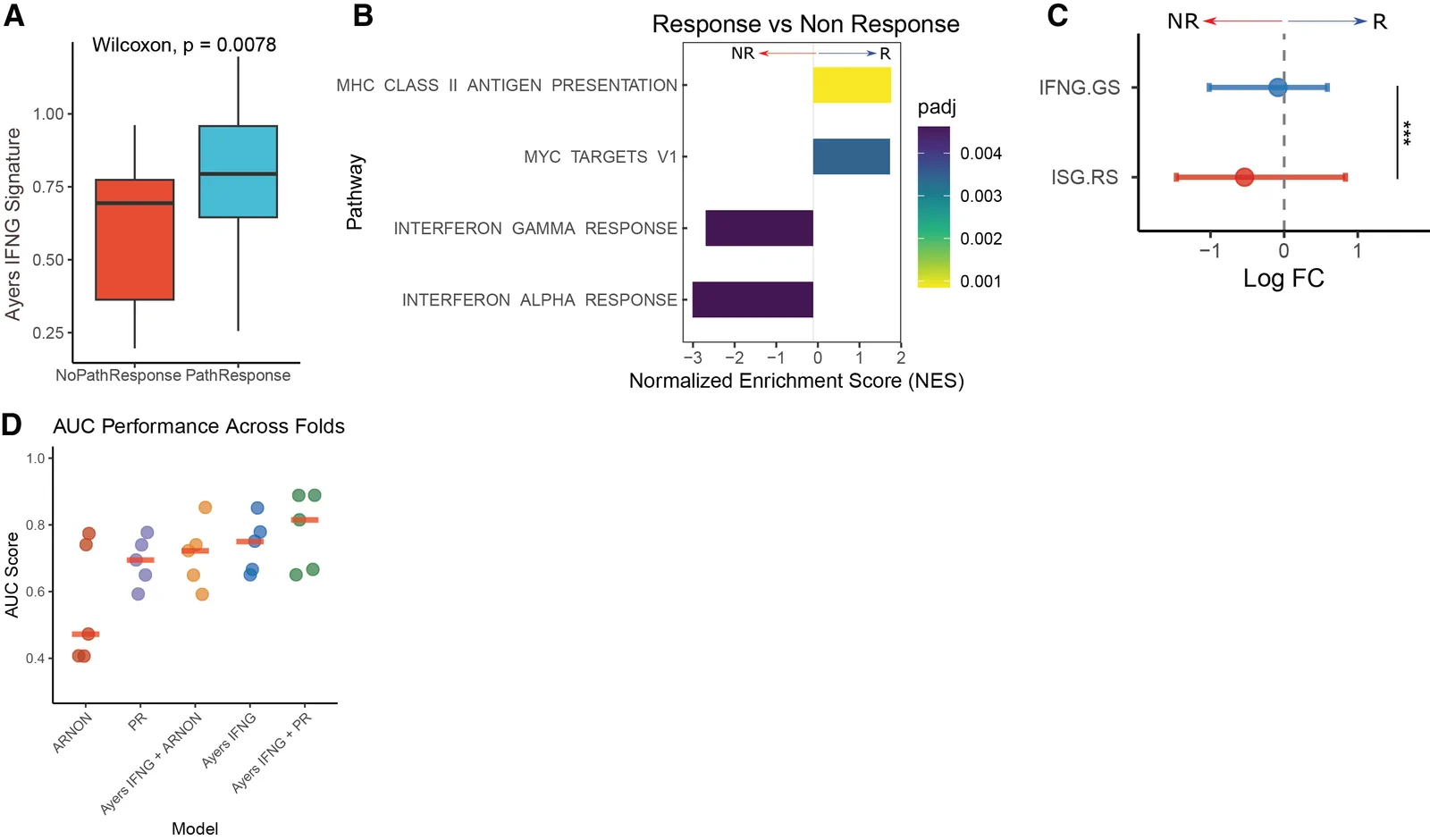
The association between interferon signaling and response in the Neoadjuvant Setting (A) The previously established IFNG-related signature^4^ scored in bulk tumors from responders and non-responders (*n* = 63). (B) The enriched pathways between responders and non-responders using the cancer cell-dependent model. The Hallmark and Reactome databases were used, applying an FDR threshold of 0.05. (C) The fold change values, identified with the cancer cell-dependent model, for interferon-related genes that were previously identified^42^ as enriched in cancer cells (ISG.RS) or immune cells (IFNG.GS) (∗*p* < 0.05, ∗∗∗*p* < 0.001). A Wilcoxon rank-sum test was used. (D) AUC values from response prediction models evaluated using 5-fold cross-validation. Each dot represents the AUC from an individual fold; the red line indicates the median AUC across all folds.

Neoadjuvant ICB is an emerging standard of care for resectable stage III melanoma.^47^^,^^48^ To investigate the role of interferon signaling in this context, we analyzed bulk RNA-seq data from the OpACIN-neo trial, which included 63 patients with resectable stage III melanoma treated with neoadjuvant ipilimumab and nivolumab (Table S12).^16^ First, we replicated the association between the bulk expression level of the previously established IFNG-related signature^4^ and pathological response (major or partial) (Figure 6A). As pathological response predicts relapse-free survival in this dataset,^16^^,^^48^ we expect pathological response to most closely resemble DR patients in the neoadjuvant setting, whereas pathological non-responders (pNRs) will be most similar to primary resistance tumors.

Using the cancer cell-dependent model, we compared responders with pNRs. Consistent with observations from the HMF and Liu datasets comparing DR and resistance patients, pNRs exhibited downregulation of melanocyte-associated genes, including *RAB27B* (FDR = 0.056), a key regulator of melanosome trafficking, and *CAPG*, a constituent protein of melanosomes (FDR <0.01) (Table S13).^49^^,^^50^ At the pathway level, pNRs upregulated IFNG and IFNA response while downregulating MYC targets and MHC class II compared to responders (Figure 6B) (Table S14), consistent with previous observations. Furthermore, fold change values from the cancer cell-dependent model revealed that ISG.RS genes were elevated in pNRs compared to responders and were significantly upregulated compared to IFNG.GS genes (*p* < 0.05) (Figure 6C). Collectively, these findings suggest that dedifferentiation—characterized by downregulation of MYC targets and the selective upregulation of interferon-related genes—is associated with resistance in both neoadjuvant and metastatic treatment settings.

Finally, we evaluated the performance of our previously described primary resistance signature (Table S10) in distinguishing responders from pNRs and compared it to a predictive cancer cell-intrinsic immune exclusion program from Jerby-Arnon et al.^21^ Using a logistic regression model with 5-fold cross-validation, we analyzed each signature individually and in combination with the previously established IFNG-related gene signature,^4^ which has previously demonstrated predictive performance in this patient cohort.^16^ The primary resistance signature alone achieved a median AUC of 0.69, outperforming the Jerby-Arnon signature (median AUC = 0.47) (Figure 6D). Notably, combining the primary resistance signature with the established IFNG-related signature (baseline AUC = 0.75) improved predictive performance to a median AUC of 0.82, demonstrating the complementary value of integrating cancer cell-dependent resistance signatures with TME immune activation status.

### The DR and AR expression programs exist on distinct genetic backgrounds

**Figure 7:**
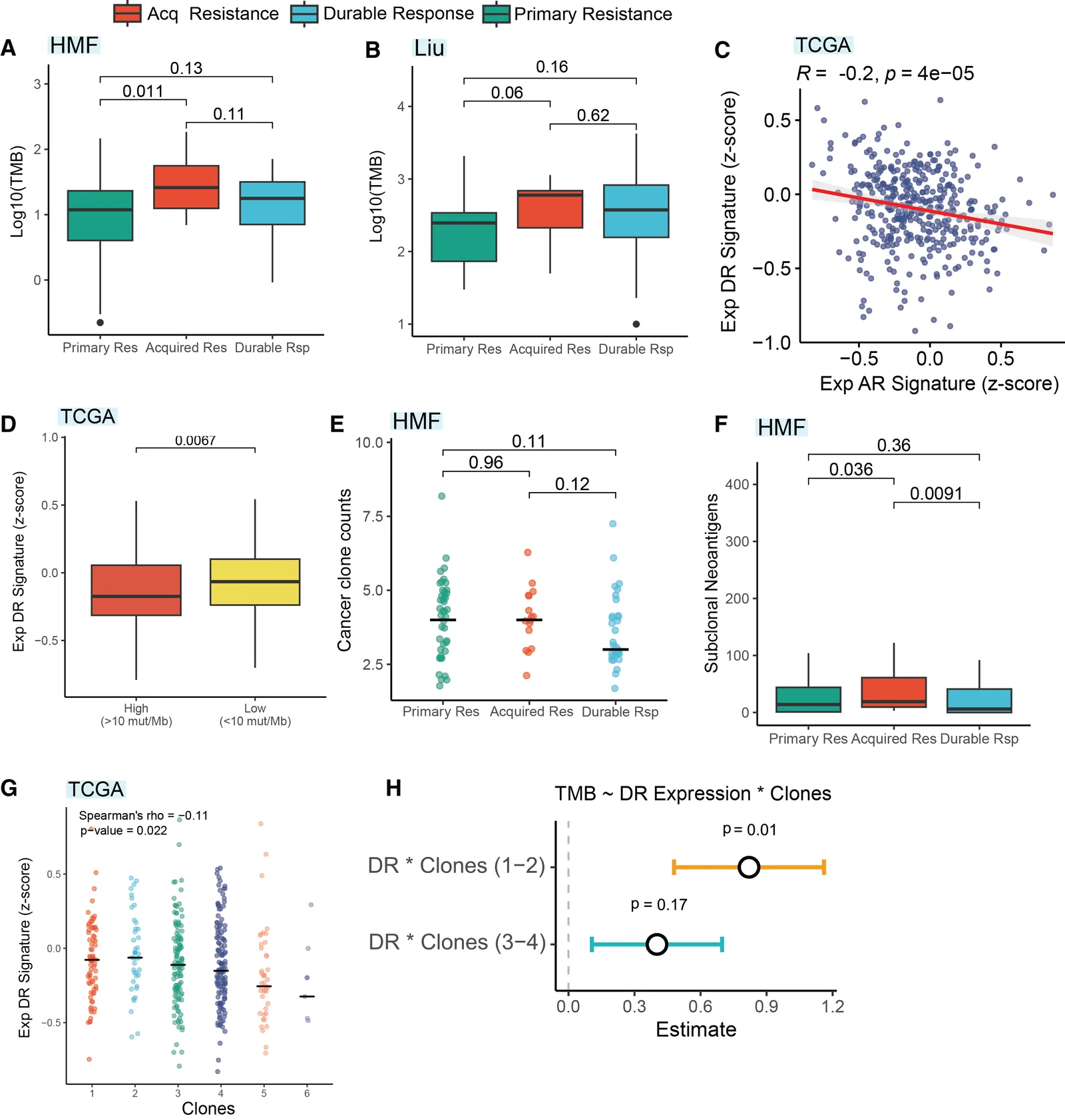
The genetic backgrounds associated with the cancer cell-dependent expression profiles in AR and DR patients (A and B) TMB estimates for the duration of response groups in the HMF and Liu et al. datasets, respectively. Patients with matched WGS and RNA-seq data were included (*n* = 96). (C) The correlation between the inferred *Z* scores for genes upregulated in DR and AR patients in TCGA samples. (D) The expression of genes upregulated in DR patients in TCGA samples with either high or low TMB. A threshold of 10 nonsynonymous mutations was used to dichotomize patients. (E) The number of cancer clones in the duration of response groups. (F) The number of subclonal neoantigens in the duration of response groups. (G) The expression of genes upregulated in DR patients in TCGA samples according to the number of clones in the tumor. (H) The relationship between the DR signature and TMB in TCGA samples, grouped by clone counts. The 5–6 clone group is the reference, with *p* values calculated relative to this group. Statistical comparisons in (A), (B), and (D–F) were performed using a two-sided Wilcoxon rank-sum test.

High tumor mutational burden (TMB) and neoantigen load are associated with response to ICB.^51^ However, in melanoma, an excessively high TMB has been linked to reduced OS.^52^ The relationship between TMB and response durability has not been explored. In both the HMF and Liu datasets, patients with primary resistance had the lowest TMB, while patients with AR had a numerically higher TMB than DR patients (Figures 7A and 7B) (*p* = 0.11 and 0.62, respectively).

To improve statistical power, we leveraged the large samples sizes provided by TCGA. Using CIBERSORT *high resolution*, we inferred cancer cell-intrinsic expression profiles from skin cutaneous melanoma samples.^53^ We then computed signature scores (*Z* scores) for genes identified by the cancer cell-dependent model as upregulated in DR and AR patients from the HMF dataset (detailed in methods). Notably, the DR and AR gene signatures were inversely correlated (R = −0.2, *p* < 0.05; Figure 7C), suggesting that these transcriptional programs are not co-expressed. Furthermore, TCGA samples with a low TMB (<10 mut/Mb) had increased expression of the DR signature compared to samples with a high TMB (*p* < 0.01) (Figure 7D), consistent with the lower TMB observed in DR relative to AR patients.

Despite the trend toward elevated TMB in AR compared to DR patients, no difference in clonal neoantigen burden was observed (Figure S2I). However, DR patients in the HMF dataset had numerically fewer tumor clones and significantly fewer subclonal neoantigens compared to AR patients (*p* = 0.12 and *p* < 0.01, respectively) (Figures 7E and 7F). Furthermore, TCGA tumors with fewer clones exhibited significant upregulation of the DR signature (*p* < 0.05) (Figure 7G), in agreement with the lower intra tumor heterogeneity observed in DR patients. In tumors with low clonal diversity (1–2 clones), the correlation between TMB and the DR signature was significantly stronger than in clonally diverse tumors (5–6 clones) (*p* = 0.01; Figure 7H), suggesting that the low TMB observed in DR patients reflects high mutational burden within clonally homogeneous tumors. In contrast, the elevated TMB in AR patients may reflect increased clonal diversification, which can impair effective immune surveillance.^54^ These observations are consistent with recent reports describing antigenic cancer persister cells that survive prolonged cytotoxic T cell attack despite remaining immunogenic, thereby providing a reservoir from which resistant clones can emerge.^55^

## Discussion

The treatment of metastatic melanoma has been revolutionized by the advent of ICB, the mainstay being combination anti-PD1 and anti-CTLA4 therapy.^2^ However, despite the high initial response rate to first-line nivolumab plus ipilimumab, 24% of patients develop acquired resistance, while only 34% achieve durable responses.^56^ Transient responders still endure significant toxicity with limited second-line options, making early identification essential for alternative therapies.

Building on extensive research, we confirmed that deconvolution-derived estimates of immune cell infiltration correlate with initial ICB response.^4^^,^^13^^,^^22^^,^^57^ However, among responders, immune cell infiltration demonstrated an inconsistent relationship with durable response in the HMF (no association) and Liu datasets (decreased in durable responders). Therefore, bulk-level quantification of immune cell biomarkers cannot reliably predict the duration of ICB response.

Given the paucity of suitable scRNA-seq datasets and limitations of bulk RNA-seq, we employed a regression-based approach using longitudinal ICB response data to identify cancer cell-dependent expression programs associated with durable responses. Adapting established deconvolution principles,^9^^,^^11^ we leveraged variations in tumor purity to delineate cancer cell-dependent signals. We validated our approach using scRNA-seq data, where cancer cell transcriptomes could be defined with high confidence. Our approach outperforms standard univariate methods at identifying cancer cell-dependent expression and shows robustness in low purity tumors—a common characteristic of highly immune-infiltrated melanoma.^18^

IFNG is a potent cytokine central to anti-tumor immune responses.^4^ In melanoma cells, it can also drive dedifferentiation, a cellular state commonly associated with resistance to targeted therapy and immunotherapy.^30^^,^^38^ We found that cancer cells from pre-treatment tumors that initially respond, but later acquire resistance to first-line ICB, were associated with increased interferon signaling, reduced levels of MYC activity, and a dedifferentiated phenotype at baseline. Studies have demonstrated that on-treatment samples from patients responding to ICB also experience interferon-induced dedifferentiation, characterized by downregulation of key transcription factors such as *MITF* and *MYC*.^31^^,^^58^ This suggests that interferon-induced dedifferentiation is a sign of inflammation and as such both a marker of response and a precursor to acquired resistance, depending on its timing. We hypothesize that when melanoma cells are exposed to interferon prior to ICB without their complete clearance, dedifferentiation occurs and mechanisms of immune escape are acquired. For example, we observed downregulation of melanocyte-associated proteins, a trend toward increased *PDL1* expression, and a significant reduction in MHC class II pathway induction by interferon in AR patients.

The cancer cell-dependent expression programs from DR and AR patients coincided with differences in the T cell compartment. For example, T cell levels in DR patients were associated with a cytotoxic and memory T cell phenotype and were more correlated with interferon signaling, response to chemokines, and integrin interactions compared to AR patients. In AR patients, reduced cancer cell-dependent expression of melanocyte proteins associated with cellular dedifferentiation may contribute to lower enrichment of memory T cells, consistent with evidence that resident memory T cells are shared between tumors and vitiligo-affected skin in exceptional responders.^59^

Primary resistant patients exhibited an immune-cold phenotype, low PD-L1 expression, high CIN, elevated HERV expression, and a bias toward NFKB-driven interferon signaling. As both CIN and HERVs can activate cell-intrinsic interferon responses, this may contribute to the increased interferon signaling associated with cancer cells from these patients. However, the lack of upregulation in cytosolic DNA and RNA sensors suggests that post-transcriptional mechanisms, as previously reported,^60^ may be responsible.

Acquired resistance tumors exhibited higher TMB, more subclones, and a greater burden of subclonal neoantigens compared to durable responders, suggesting clonal complexity and intratumoral heterogeneity (ITH). This ITH may facilitate tumor progression despite exposure to both pre-treatment and ICB-induced interferon, potentially contributing to chronic interferon signaling.

Despite the distinct phenotypes of primary and acquired resistance, both exhibited increased cancer cell-dependent IFNG and IFNA response before treatment. This suggests that chronic interferon exposure may result in inflammatory memory in tumor cells,^6^ driving mechanisms of immune escape that ICB can, at most briefly, overcome. Therapies that counteract this inflammatory response might be needed to reverse this state and sensitize tumors to immune killing.

Previous efforts to elucidate mechanisms of acquired resistance to ICB have relied on modestly sized, heterogeneously treated patient cohorts^6^^,^^8^^,^^40^^,^^42^^,^^61^ and preclinical models to identify cancer cell-intrinsic mechanisms of resistance. In contrast, we identified candidate resistance mechanisms in large metastatic melanoma cohorts receiving first-line ICB—the current standard of care. These findings hold promise for assigning patients that will experience transient responses for alternative or combination treatments, thereby limiting unnecessary ICB-related toxicities and expanding the population that may ultimately achieve durable disease control. Finally, the transcriptomic features associated with acquired resistance in these baseline samples mirrored changes observed in on-treatment samples, underscoring the potential value of examining ICB responses as a dynamic, evolving process.

### Limitations of the study

Our study has limitations that should be considered. First, while our model deconvolves cancer cell signals from TME confounders, the microenvironment’s complexity precludes accounting for every component. Consequently, we cannot definitively assign the precise cellular origin of all observed expression changes. Second, the median PFS follow-up for durable responders was 20 months in the HMF dataset. Since PFS curves plateau at 3 years post-ICB in metastatic melanoma, longer follow-up would reduce potential misclassification of late-relapse patients as durable responders. Finally, this study represents a hypothesis generating clinical discovery analysis designed to identify candidate features underlying response duration in large-scale retrospective cohorts. Experimental validation is needed to confirm the causal mechanisms responsible for these associations, and prospective cohorts are essential to test signature performance in a clinical setting.

## Resource availability

### Lead contact

Further information and requests for resources should be directed to and will be fulfilled by the lead contact, Samra Turajlic (samra.turajlic@crick.ac.uk).

### Materials availability

This study did not generate new unique reagents.

### Data and code availability

• The HMF bulk RNA-seq and genomic data are available at https://www.hartwigmedicalfoundation.nl/en/data/data-access-request/. Processed somatic mutational profiles can be accessed at https://github.com/UMCUGenetics/Genetic-Immune-Escape/. The Liu et al. dataset was obtained from https://github.com/vanallenlab/schadendorf-pd1, while the OpACIN-neo dataset was accessed from EGAS00001004833. The scRNA-seq datasets from Pozniak et al. and Jerby-Arnon et al. are available at https://doi.org/10.48804/GSAXBN and GSE115978, respectively. RNA-seq data for IFNG-treated melanoma cell lines were accessed from GSE80829. The bulk RNA-seq compendium is available in the supplementary data of Bagaev et al.^22^ The SKCM TCGA data can be retrieved from https://www.cbioportal.org/study/summary?id=skcm_tcga. Estimates of cancer clones in TCGA samples were obtained from https://github.com/bioinf-dev/Wolf_et_al_Cell_2019.
• The source code and data for reproducing analyses and figures are available at https://github.com/DonaghEgan/Cancer_Expression_DR_AR and https://doi.org/10.5281/zenodo.19898253.
• Any additional information required to reanalyze the data reported in this work paper is available from the lead contact upon request.

## Acknowledgments

This was conducted with the financial support of Precision Oncology Ireland, a Consortium of five Irish Universities, six Irish Charities, and seven Industry Partners, which is funded by the Science Foundation Ireland Strategic Partnership Programme, under grant number (18/SPP/3522). Additional support was provided by 10.13039/100010438The Francis Crick Institute, which receives its core funding from 10.13039/501100000289Cancer Research UK (FC010110), the UK 10.13039/501100000265Medical Research Council (FC010110), and the 10.13039/100010269Wellcome Trust (FC010110), and by 10.13039/501100000272NIHR BRC at the Royal Marsden Hospital and Institute of Cancer Research (A109). S.T. is funded by The Francis Crick Institute and the Wellcome Trust (FC10988). I.L. is funded by the Bjorn Saven Fellowship Fund. S.T. is also funded by the 10.13039/501100000272National Institute for Health Research (NIHR) Biomedical Research Center at the Royal Marsden Hospital and Institute of Cancer Research (grant reference number A109), the 10.13039/100016916Royal Marsden Cancer Charity, the 10.13039/501100000833Rosetrees Trust (grant reference number A2204), Ventana Medical Systems Inc. (grant reference numbers 10467 and 10530), the National Institute of Health (U01 CA247439), 10.13039/100005190Melanoma Research Alliance (Award Ref no 686061), the UxS Department of Defense (award no W81XWH-22-1-0652) and 10.13039/100013920VHL Alliance (grant no PRJ_20450), and the Office of Life Sciences and the 10.13039/501100000265UK Medical Research Council (grant reference number MR/Z505158/1).

## Author contributions

Conceptualization, D.E., I.L., and S.T.; methodology, D.E., I.L., and B.S.; formal analysis, D.E., I.L., B.S., and R.F.; data curation, D.E., B.S., I.L., and S.T.; writing – original draft, D.E. and I.L.; writing – review & editing, B.S. and S.T.; visualization, D.E. and I.L.; supervision, I.L. and S.T.; funding, W.K., D.B., and S.T.; project administration, S.T.

## Declaration of interests

S.T. reports speaking fees from Roche, AstraZeneca, Novartis, and Ipsen and has filed the following patents: indel mutations as a therapeutic target and predictive biomarker (PCTGB2018/051892 and PCTGB2018/051893) and clear-cell renal cell carcinoma biomarkers (P113326GB).

## STAR★Methods

### Key resources table

**Table:** 

REAGENT or RESOURCE	SOURCE	IDENTIFIER
**Deposited data**		

Hartwig Medical Foundation (HMF) dataset	Hartwig Medical Foundation	https://www.hartwigmedicalfoundation.nl/en/data/data-access-request/%20
Processed Somatic Mutation Profiles (HMF)	GitHub	https://github.com/UMCUGenetics/Genetic-Immune-Escape/
Liu et al. dataset	Liu et al. (GitHub)	https://github.com/vanallenlab/schadendorf-pd1
OpACIN-neo dataset	European Genome-Phenome Archive	EGAS00001004833
Jerby-Arnon et al. scRNA-seq dataset	NCBI GEO	GSE115978
Pozniak et al. scRNA-seq dataset	RDR	https://doi.org/10.48804/GSAXBN
IFNG-treated melanoma cell lines	NCBI GEO	GSE80829
Bagaev et al. bulk RNA-seq compendium	Bagaev et al. (Publication)	https://doi.org/10.1016/j.ccell.2021.04.014
Estimates of TCGA cancer clones	Wolf et al. (GitHub)	https://github.com/bioinf-dev/Wolf_et_al_Cell_2019
TCGA RNA-seq data	cBioPortal	https://www.cbioportal.org/study/summary?id=skcm_tcga

**Software and algorithms**		

nf-core/rnaseq pipeline (v3.11.2)	nf-core	https://nf-co.re/rnaseq/3.11.2
FastQC(V0.11.9)	Babraham Bioinformatics	https://www.bioinformatics.babraham.ac.uk/projects/fastqc/
TrimGalore! (v0.6.6)	Babraham Bioinformatics	https://www.bioinformatics.babraham.ac.uk/projects/trim_galore/
STAR (v2.6.1 day/v2.7.10a)	GitHub (Alex Dobin et al.)	https://github.com/alexdobin/STAR
tximport (v1.28.0)	Bioconductor	tximport
DESeq2	Bioconductor	DESeq2
MixCR (v3.0.3)	MixCR Documentation	https://mixcr.com/
CIBERSORTx	Stanford University	https://cibersortx.stanford.edu/
EnrichR	Ma’ayan Lab	https://maayanlab.cloud/Enrichr/
fgsea (v1.26.0)	Bioconductor	fgsea
msigdbr R package	Github	msigdbr
BWA mem (v2.2.136)	BWA	https://bio-bwa.sourceforge.net/
samtools	HTSlib	https://www.htslib.org/
GATK (v4.1.8.1)	Broad Institute	https://gatk.broadinstitute.org/hc/en-us
PyClone-VI	Roth Lab/GitHub	https://github.com/Roth-Lab/pyclone-vi
nf-core/rnafusion (v3.0.2)	nf-core	https://nf-co.re/rnafusion/3.0.2/
Arriba (v2.4.0)	Arriba GitHub	https://github.com/suhrig/arriba
Monocle3	Trapnell Lab	https://cole-trapnell-lab.github.io/monocle3/
AUCell	Bioconductor	AUCell
Code Repository (analysis scripts)	Zenodo	https://doi.org/10.5281/zenodo.19898253
Code Repository (analysis scripts)	Donagh Egan/GitHub	https://github.com/DonaghEgan/Cancer_Expression_DR_AR

### Experimental model and study participant details

The Hartwig Medical Foundation (HMF) has characterized a large pan-cancer cohort (*n* = 3302), available at: https://www.hartwigmedicalfoundation.nl. We accessed 131 metastatic melanoma samples from this cohort, all collected before first-line ICB. Bulk RNA-seq data were available for 108 samples (64 male, 44 female; median age 66 years, range 26–94). Tumor purity was estimated from WGS data, and to maximize tumor cellularity, biopsies with <20% purity were excluded and the highest-purity sample was selected when multiple biopsies per patient were available, as described by Priestley et al.^62^ Information on ancestry, race, and ethnicity was not available in the source cohort. Sex was not significantly associated with duration of response group (chi-square test, *p* = 0.36, *n* = 96) or with binary response status (Fisher’s exact test, *p* = 0.33, *n* = 108). Analyses were not powered to detect sex-specific effects.

We analyzed two additional datasets from Liu et al.^17^ and the OpACIN-neo study.^16^ For the Liu dataset, raw counts were retrieved from the GitHub repository (https://github.com/vanallenlab/schadendorf-pd1), and for OpACIN-neo, FASTQ files were accessed via EGAS00001004833. Patient clinical data and tumor purity estimates were accessed from the supplementary materials of the respective publications. To ensure consistency with the HMF dataset, we excluded from the 121 patients with RNA-seq data in the Liu dataset those with prior anti-CTLA4 therapy (*n* = 47), MAPK inhibitor treatment (*n* = 7), other undefined prior treatments (*n* = 6), mixed response (*n* = 2), or inconsistent data (*n* = 2). This study is a secondary analysis of previously published and controlled-access datasets; no new human samples were collected, and all source cohorts received ethical approval and informed consent in their original studies.

### Method details

#### Defining clinical outcomes

Best objective response was defined using RECIST 1.1 criteria for the HMF and Liu datasets. In the HMF cohort, response assessment was based on overall disease burden and M-stages (M1a-d) were inferred from baseline disease sites (Table S1). A CR or PR denoted response, whilst stable disease (SD) and progressive disease (PD) denoted non-response. In both datasets, longitudinal response information was used to characterize patients as primary resistance, acquired resistance (AR), or durable response (DR). An initial response, or SD exceeding 6 months was required to be categorized as either DR or AR. Within these, patients that progressed during subsequent follow-up were classified as AR, and patients who did not were classified as DR. Patients with PD or SD not exceeding 6 months did not benefit from treatment and were classified as having primary resistance. When longitudinal response information was not available for a period exceeding 6 months, patients were not assigned to a duration of response group for downstream analysis. A total of 12 patients were not classified, resulting in 96 patients with RNA-seq data for comparisons between the duration of response groups.

In the OpACIN-neo dataset, response was classified as partial (PR) or complete pathological response (CR), with all other outcomes considered non-response. The clinical characteristics of each dataset are summarized in Tables S1, S5, and S12.

#### RNA-seq data processing

The HMF and OpACIN-neo sequencing data were analyzed using the nfcore/rnaseq pipeline (v3.11.2).^63^ First, the input FastQ files underwent quality evaluation with FastQC (0.11.9) and adapter trimming with TrimGalore! (0.6.6). Next, the reads were aligned to the human reference genome GRCh38 using STAR (2.6.1 day) and quantified with SALMON (1.10.1)^64^ The counts were imported using the tximport (1.28.0) R package.

#### Differentially expressed genes: Univariate and cancer cell-dependent model

Differentially expressed genes between responders and non-responders, and between the duration of response groups, were identified using DESeq2 with raw counts provided as input.^64^ Genes with less than 5 reads across the samples were filtered. Wald’s test with Benjamini-Hochberg (BH) correction was used to assess significance. For the univariate models, a single factor representing the patient outcome group was used.

For the cancer cell-dependent model, a multivariate generalized linear model with an interaction term was used to identify relationships between tumor purity and gene expression that depended on the duration of response groups.

For each gene g, we fit the following model:

$$\log_{2}(q)\;=\;\beta ₀\;+\;\beta ₁\cdot TP\;+\;\beta ₂\cdot DG\;+\;\Sigma ₖ\;\beta ₃ₖ\cdot Sₖ\;+\;\beta ₄\cdot (TP\;\cdot \;DG)$$

Here, q is the normalized expression of gene g, *TP* represents tumor purity, *DG* represents the duration of response groups, and *S_k_* denotes the abundance of k cell types in the stromal compartment responsible for variation between samples. We identified antigen-presenting cells, non-antigen presenting cells, endothelial and fibroblasts as sources of variation by performing PCA on the deconvolution-derived estimates of cell type abundances. *β0* represents the intercept, and each *βi* (for *i* > 0) is the log_2_ fold change per unit change in the corresponding predictor, holding the other variables constant. A significant adjusted *p* value (FDR <0.05) for the *β4* coefficient indicates an interaction effect between *TP* and *DG.* This FDR threshold was relaxed to 0.1 when identifying duration of response signatures in the HMF and Liu datasets. The model was implemented using DEseq2,^64^ with raw counts provided as input.

To identify T cell-dependent expression changes, the model was reformulated as follows:

$$\log_{2}(q)\;=\;\beta ₀\;+\;\beta ₁\cdot TCB\;+\;\beta ₂\cdot TP\;+\;\beta ₃\cdot DG\;+\;\Sigma ₖ\;\beta ₄ₖ\cdot Sₖ\;+\;\beta ₅\cdot (TCB\;\cdot \;DG)$$

Here, *TCB* represents T cell burden, and was inferred using MixCR (described below). All other terms are as defined above.

#### Quantification of TCR and ig reads in RNA-seq data

We used MixCR v3.0.3 to quantify T cell and B cells levels from transcriptomic reads, as described in Freeman et al.^15^ Briefly, we ran MixCR analyze shotgun with the starting material parameter set to RNA. We removed TCR or Ig clonotypes with rearrangements that had a stop codon or resulted in an out-of-frame sequence. The number of TCR or Ig reads were quantified by summing the clone counts of reads assigned to clonotypes. TCR alpha, beta, delta and gamma sequences were included. Rare clonotypes were aligned to both a TCR and an Ig, and we included these reads in both the TCR and the Ig counts. We normalized the TCR and Ig read counts by sample coverage, using the number of mapped reads from samtools flagstat.^65^ To create the T cell burden (TCB) and B cell burden (BCB) metrics, we computed TCB = (1+TCR read count)/(aligned reads/106) and BCB = (1+Ig read count)/(aligned reads/106).

#### TME deconvolution and comparisons

The consensus tumor microenvironment (consensusTME) cell estimation method was applied to deconvolve the TME.^12^ Gene sets specific for human skin cutaneous melanoma (SKCM) were used. Differentially abundant cell types were identified by min-max scaling the inferred abundances. The median normalized enrichment scores (NES) for each patient group were determined, and the difference was calculated by taking the logarithm base 2 ratio of the median NES. Statistical significance was calculated using a Wilcoxon rank-sum test with BH correction, and an FDR threshold of 0.05 was applied.

#### Gene set enrichment analysis

GSEA was conducted using the fgsea (version 1.26.0) package in R. The Hallmark and Reactome gene sets from MSigDB were accessed through the R package msigdbr. T cell gene signatures from Sade Feldman et al.^13^ were made available in the supplementary data. A pre ranked gene list according to the Wald statistic from DESeq2 was provided as input. An FDR threshold of 0.05 was applied to identify significant pathways. For specific pathways of interest, such as MHC class I and II from the Reactome database, significance was assessed using a nominal *p*-value threshold of 0.05.

#### Model validation

A bulk RNA-seq dataset of sorted cell populations was accessed from Bagaev et al.^22^ Gene expression values were *Z* score normalized, and the median expression of significant genes (FDR <0.1) from the univariate and cancer cell-dependent model were compared for the cell types present.

For the scRNA-seq validation dataset, transcripts per million (TPM) values were accessed through NCBI’s Gene Expression Omnibus (GEO) under accession number GSE115978.^21^ The proportion of each cell type in a sample was calculated, and then hierarchical clustering was performed according to the stromal compartment, with two clusters being identified. Using the Seurat pipeline, differentially expressed genes were identified between the malignant cells in both clusters (|logFC| > 0.5; FDR <0.05), establishing a ground truth.^66^ Pseudo bulk profiles were created by summing the counts of all cells belonging to a tumor, and DEGs between both clusters were determined using a standard univariate approach, or with the cancer cell-dependent model. The log fold change values were sampled from the ground truth and compared to the results obtained from both the univariate and cancer cell-dependent models using Pearson’s correlation coefficient. This protocol was then repeated after the removal of 25% of malignant cells in silico. The correlation coefficients generated for each model were compared using a Wilcoxon rank-sum test.

#### Testing the previously established IFNG-related signature

A 10 gene signature (IFNG, STAT1, CCR5, CXCL9, CXCL10, CXCL11, IDO1, PRF1, GZMA, and HLA-DRA) published by Ayers et al.^4^ was scored in the count matrices of the HMF, Liu, and OpACIN-neo datasets using the gsva function in R (method = “ssgsea”). The signature scores were compared between response groups using a Wilcoxon rank-sum test.

#### IFNG treatment and differentiation analysis in melanoma cell lines

The raw RNA-seq counts for the IFNG-treated cell lines were accessed at GSE80829.^31^ The counts were normalized using DESeq2. The MYC targets pathway from the Hallmarks database and the MHC class II pathway from Reactome were scored in differentiated and dedifferentiated human melanoma cell lines, as defined by the original authors, using single sample GSEA (method = “*Z* score”). The M308 cell line was excluded from MHC class II analyses due to splice site mutations in JAK1 and JAK2, which function upstream of MHC class II. Statistical significance was assessed using a two-sided *t* test following a Shapiro-Wilk test to confirm normality.

#### Gene signature generation

We created gene signatures for DR and AR patients from DEGs identified between these groups in the HMF and Liu datasets. A parallel analysis comparing primary resistance and DR patients was used to generate a primary resistance signature. All signatures were derived from the cancer cell-dependent model and an FDR threshold of <0.1 was applied.

#### Pseudotime analysis

The Seurat object from the Pozniak et al. scRNA-seq dataset was accessed at https://doi.org/10.48804/GSAXBN.^32^ Cells annotated as “Mitochondrial (low quality)“, ”Patient specific A“, and ”Patient specific B” were removed due to a lack of biological relevance. Pseudotime analysis was conducted with monocle3,^67^ using the UMAP reduction provided by the original authors as input, and Mesenchymal cells as the root node. The gene signatures for AR and DR patients, the AXL signature from Tirosh et al., the ISG.RS and IFNG.GS from Benci et al. were quantified using AUCell.^39^^,^^42^^,^^68^ To mitigate sparsity effects, signature scores were calculated as a weighted average of k-nearest neighbors using UCell.^69^

#### Analysis of OpACIN-neo

Raw counts were filtered and normalized using DESeq2.^64^ The cancer cell-dependent model was implemented as described above, but with responders and pNRs used as response groups. Gene signatures were scored using the GSVA R package (method = “ssgsea”). Logistic regression models were then trained to predict clinical response (responders versus pNRs) using either individual signatures or combinations of signatures. To assess predictive performance, we carried out stratified 5-fold cross-validation, while preserving the original proportion of responders and pNRs within each fold. Each fold served once as the test set, with the remaining 4-folds used for training. Model performance was evaluated using the area under the ROC curve, and the median AUC across the 5-folds was reported.

#### CIBERSORT high resolution in TCGA samples

A CIBERSORTx signature matrix was created using count data from Tirosh et al.,^39^ which is provided as an example dataset on the CIBERSORTx website (https://cibersortx.stanford.edu/runcibersortx.php). This signature matrix, combined with a skin cutaneous melanoma cohort from The Cancer Genome Atlas (TCGA),^53^ served as inputs for the Impute Cell Expression module, executed in high resolution mode. The top 1,000 upregulated genes in cancer cells from DR and AR patients were imputed, as the method’s capacity is 1,000 genes. The remaining parameters were kept at their default settings, and the resulting matrix was log2 transformed. The signature scores for the genes upregulated in DR and AR patients were calculated by scaling the gene expression values, and calculating the median for each sample. As the accuracy of CIBERSORTx depends on the number of tumors profiled,^20^ high-resolution mode was used when sample size was sufficient or when the number of genes to be imputed was low.

#### RNA fusion calling

To identify RNA fusions, the nf-core^63^ rnafusion^70^ pipeline version 3.0.2 was used. In short, RNA-seq reads were aligned to the GRCh38 reference genome with STAR version 2.7.10a^71^ Arriba version 2.4.0^72^ was used to detect gene fusions, which were filtered by the “High” confidence annotation from Arriba.

#### HERV expression quantification

Transcriptomic reads were aligned to the human reference genome (GRCh38) using STAR (v2.6.1 day).^71^ Resulting SAM files were converted to BAM format and collated by read name using SAMtools (v1.1). Each sample was then processed with the telescope assign command using the “HERV_rmsk.hg38.v2” annotation, a reference of 60 HERV families.^73^ To identify HERV loci that varied significantly across duration of response groups, we performed a likelihood ratio test using DESeq2, including tumor purity as a covariate (FDR <0.05). For each significant locus, we determined which group exhibited the highest mean expression. To test whether upregulated HERVs were enriched in specific groups beyond random expectation, we applied a binomial test comparing the observed distribution against the null hypothesis of equal distribution across all groups.

#### Transcription factor enrichment

To evaluate possible differences between the IFNG response pathway in AR versus PR tumors, we analyzed the leading-edge genes exclusive to each clinical phenotype in the HMF and Liu cohorts. We performed initial enrichments using EnrichR^74^ and found the TRRUST Transcription Factors 2019 set results to be different between duration of response groups but consistent across the cohorts. We confirmed the results using TRRUST version 2 to identify the key transcription factors regulating the different human gene sets.^75^

#### Tumor mutational burden and intra-tumor heterogeneity analysis

TMB estimates were obtained from the supplementary data of Liu et al. and Martínez-Jiménez et al. for the HMF cohort, and from cBioPortal (https://www.cbioportal.org/) for the TCGA cohort.^17^^,^^62^ The number of cancer clones in TCGA samples was accessed from https://github.com/bioinf-dev/Wolf_et_al_Cell_2019.^76^ A two-sided Wilcoxon rank-sum test was used to assess significance. To calculate the number of clones in the HMF cohort, we first reprocessed the provided cram files. Briefly, we extracted the reads with samtools collate and samtools fastq,^65^ and mapped them to the hg38 reference genome using BWA mem 2.2.1^77^ as implemented in nf-core Sarek 3.1.1.^78^ We then used GATK v4.1.8.1 Mutect2^79^ to call somatic mutations and required that mutations passed all filters and had at least 4 alternative reads and a read orientation bias ROQ of 20. We used Battenberg to call purity and ploidy,^80^ and manually reviewed solutions and rerun them where necessary. We used the copy number segments, mutation allelic counts and purity as inputs for PyClone-VI to identify mutation clusters with different cancer cell fractions.^81^ All analyses were performed on patients with matched bulk RNA-seq and WGS.

### Quantification and statistical analysis

All statistical analyses were conducted in R (version 4.2.1). Detailed information for each analysis, including the statistical tests used, sample sizes, and significance thresholds, is provided in the figure legends and the Results section. Unless otherwise specified, boxplots display the median as the center line, with the interquartile range (IQR) as the box limits and 1.5× IQR as whiskers. Group comparisons were performed using two-sided Wilcoxon rank-sum tests unless normality was confirmed by a Shapiro-Wilk test, in which case a two-sided *t* test was used. Multiple testing correction was applied using the Benjamini-Hochberg method, with FDR thresholds specified in each analysis. Statistical significance is denoted as: ∗*p* < 0.05; ∗∗*p* < 0.01; ∗∗∗*p* < 0.001; ns, not significant.

## Supplemental information

Document S1. Figures S1 and S2 and Tables S1, S5, and S12

Table S2. An ANOVA test quantifying the variance of deconvolution-derived cell types and tumor purity across sample sites in the HMF dataset

Table S3. Tumor purity, B cell burden, and T cell burden estimates across the response and duration-of-response groups in the HMF dataset

Table S4. Gene set enrichment analysis (fgsea) comparing responders and non-responders in the HMF dataset using Hallmark gene sets

Table S6. Differentially expressed genes between DR and AR patients in the HMF dataset using the cancer-cell-dependent model

Table S7. Differentially expressed genes between DR and AR patients in the Liu et al. dataset using the cancer-cell-dependent model

Table S8. Enriched Hallmark pathways between DR and AR patients in the HMF dataset using the cancer-cell-dependent model

Table S9. Enriched Hallmark pathways between DR and AR patients in the Liu et al. dataset using the cancer-cell-dependent model

Table S10. Gene signatures identified by comparing AR, DR, and PR patients with the cancer-cell-dependent model

Table S11. Enriched pathways from Reactome and Benci et al, between DR and AR patients in the HMF dataset using the T-cell-dependent model

Table S13. Differentially expressed genes between pathological responders and pathological non-responders in the OpACIN-neo dataset using the cancer-cell-dependent model

Table S14. Enriched Hallmark pathways between pathological responders and pathological non-responders in the OpACIN-neo dataset using the cancer-cell-dependent model

Document S2. Article plus supplemental information

## References

[1] Postow M.A., Callahan M.K., Wolchok J.D. (2015). Immune Checkpoint Blockade in Cancer Therapy. J. Clin. Orthod..

[2] Wolchok J.D., Chiarion-Sileni V., Rutkowski P., Cowey C.L., Schadendorf D., Wagstaff J., Queirolo P., Dummer R., Butler M.O., Hill A.G. (2025). Final, 10-Year Outcomes with Nivolumab plus Ipilimumab in Advanced Melanoma. N. Engl. J. Med..

[3] Litchfield K., Reading J.L., Puttick C., Thakkar K., Abbosh C., Bentham R., Watkins T.B.K., Rosenthal R., Biswas D., Rowan A. (2021). Meta-analysis of tumor- and T cell-intrinsic mechanisms of sensitization to checkpoint inhibition. Cell.

[4] Ayers M., Lunceford J., Nebozhyn M., Murphy E., Loboda A., Kaufman D.R., Albright A., Cheng J.D., Kang S.P., Shankaran V. (2017). IFN-γ–related mRNA profile predicts clinical response to PD-1 blockade. J. Clin. Investig..

[5] Haas L., Elewaut A., Gerard C.L., Umkehrer C., Leiendecker L., Pedersen M., Krecioch I., Hoffmann D., Novatchkova M., Kuttke M. (2021). Acquired resistance to anti-MAPK targeted therapy confers an immune-evasive tumor microenvironment and cross-resistance to immunotherapy in melanoma. Nat. Cancer.

[6] Qiu J., Xu B., Ye D., Ren D., Wang S., Benci J.L., Xu Y., Ishwaran H., Beltra J.C., Wherry E.J. (2023). Cancer cells resistant to immune checkpoint blockade acquire interferon-associated epigenetic memory to sustain T cell dysfunction. Nat. Cancer.

[7] Memon D., Schoenfeld A.J., Ye D., Fromm G., Rizvi H., Zhang X., Keddar M.R., Mathew D., Yoo K.J., Qiu J. (2024). Clinical and molecular features of acquired resistance to immunotherapy in non-small cell lung cancer. Cancer Cell.

[8] Lim S.Y., Shklovskaya E., Lee J.H., Pedersen B., Stewart A., Ming Z., Irvine M., Shivalingam B., Saw R.P.M., Menzies A.M. (2023). The molecular and functional landscape of resistance to immune checkpoint blockade in melanoma. Nat. Commun..

[9] Shen-Orr S.S., Tibshirani R., Khatri P., Bodian D.L., Staedtler F., Perry N.M., Hastie T., Sarwal M.M., Davis M.M., Butte A.J. (2010). Cell type–specific gene expression differences in complex tissues. Nat. Methods.

[10] Chu T., Wang Z., Pe’er D., Danko C.G. (2022). Cell type and gene expression deconvolution with BayesPrism enables Bayesian integrative analysis across bulk and single-cell RNA sequencing in oncology. Nat. Cancer.

[11] Ghoshdastider U., Rohatgi N., Mojtabavi Naeini M., Baruah P., Revkov E., Guo Y.A., Rizzetto S., Wong A.M.L., Solai S., Nguyen T.T. (2021). Pan-Cancer Analysis of Ligand–Receptor Cross-talk in the Tumor Microenvironment. Cancer Res..

[12] Jiménez-Sánchez A., Cast O., Miller M.L. (2019). Comprehensive Benchmarking and Integration of Tumor Microenvironment Cell Estimation Methods. Cancer Res..

[13] Sade-Feldman M., Yizhak K., Bjorgaard S.L., Ray J.P., de Boer C.G., Jenkins R.W., Lieb D.J., Chen J.H., Frederick D.T., Barzily-Rokni M. (2018). Defining T Cell States Associated with Response to Checkpoint Immunotherapy in Melanoma. Cell.

[14] Poggi A., Zocchi M.R. (2022). Natural killer cells and immune-checkpoint inhibitor therapy: Current knowledge and new challenges. Mol. Ther. Oncolytics.

[15] Freeman S.S., Sade-Feldman M., Kim J., Stewart C., Gonye A.L., Ravi A., Arniella M.B., Gushterova I., LaSalle T.J., Blaum E.M. (2022). Combined tumor and immune signals from genomes or transcriptomes predict outcomes of checkpoint inhibition in melanoma. Cell Rep. Med..

[16] Rozeman E.A., Hoefsmit E.P., Reijers I.L.M., Saw R.P.M., Versluis J.M., Krijgsman O., Dimitriadis P., Sikorska K., van de Wiel B.A., Eriksson H. (2021). Survival and biomarker analyses from the OpACIN-neo and OpACIN neoadjuvant immunotherapy trials in stage III melanoma. Nat. Med..

[17] Liu D., Schilling B., Liu D., Sucker A., Livingstone E., Jerby-Arnon L., Zimmer L., Gutzmer R., Satzger I., Loquai C. (2019). Integrative molecular and clinical modeling of clinical outcomes to PD1 blockade in patients with metastatic melanoma. Nat. Med..

[18] Aran D., Sirota M., Butte A.J. (2015). Systematic pan-cancer analysis of tumour purity. Nat. Commun..

[19] Zaitsev K., Bambouskova M., Swain A., Artyomov M.N. (2019). Complete deconvolution of cellular mixtures based on linearity of transcriptional signatures. Nat. Commun..

[20] Newman A.M., Steen C.B., Liu C.L., Gentles A.J., Chaudhuri A.A., Scherer F., Khodadoust M.S., Esfahani M.S., Luca B.A., Steiner D. (2019). Determining cell type abundance and expression from bulk tissues with digital cytometry. Nat. Biotechnol..

[21] Jerby-Arnon L., Shah P., Cuoco M.S., Rodman C., Su M.J., Melms J.C., Leeson R., Kanodia A., Mei S., Lin J.R. (2018). A Cancer Cell Program Promotes T Cell Exclusion and Resistance to Checkpoint Blockade. Cell.

[22] Bagaev A., Kotlov N., Nomie K., Svekolkin V., Gafurov A., Isaeva O., Osokin N., Kozlov I., Frenkel F., Gancharova O. (2021). Conserved pan-cancer microenvironment subtypes predict response to immunotherapy. Cancer Cell.

[23] Kemp E.H., Waterman E.A., Hawes B.E., O'Neill K., Gottumukkala R.V.S.R.K., Gawkrodger D.J., Weetman A.P., Watson P.F. (2002). The melanin-concentrating hormone receptor 1, a novel target of autoantibody responses in vitiligo. J. Clin. Investig..

[24] Tsurusaki Y., Koshimizu E., Ohashi H., Phadke S., Kou I., Shiina M., Suzuki T., Okamoto N., Imamura S., Yamashita M. (2014). De novo SOX11 mutations cause Coffin–Siris syndrome. Nat. Commun..

[25] Tao D.-Y., Niu H.H., Zhang J.J., Zhang H.Q., Zeng M.H., Cheng S.Q. (2022). Short stature and melanocytic nevi in a girl with ARID1B-related Coffin-Siris syndrome: a case report. BMC Pediatr..

[26] Tchanque-Fossuo C.N., Dahle S.E., Kiuru M., Isseroff R.R. (2019). Vitiligo and melanocytic nevi: New findings in Coffin-Siris syndrome associated with ARID1 germline mutation. JAAD Case Rep..

[27] Khazanchi R., Ronspies C.A., Smith S.C., Starr L.J. (2019). Patient with anomalous skin pigmentation expands the phenotype of ARID2 loss-of-function disorder, a SWI/SNF-related intellectual disability. Am. J. Med. Genet..

[28] Li M.-Y., Flora P., Pu H., Bar C., Silva J., Cohen I., Galbo P.M., Liu H., Yu X., Jin J. (2021). UV-induced reduction in Polycomb repression promotes epidermal pigmentation. Dev. Cell.

[29] Strollo R., Ponchel F., Malmström V., Rizzo P., Bombardieri M., Wenham C.Y., Landy R., Perret D., Watt F., Corrigall V.M. (2013). Autoantibodies to Posttranslationally Modified Type II Collagen as Potential Biomarkers for Rheumatoid Arthritis. Arthritis Rheum..

[30] Landsberg J., Kohlmeyer J., Renn M., Bald T., Rogava M., Cron M., Fatho M., Lennerz V., Wölfel T., Hölzel M., Tüting T. (2012). Melanomas resist T-cell therapy through inflammation-induced reversible dedifferentiation. Nature.

[31] Kim Y.J., Sheu K.M., Tsoi J., Abril-Rodriguez G., Medina E., Grasso C.S., Torrejon D.Y., Champhekar A.S., Litchfield K., Swanton C. (2021). Melanoma dedifferentiation induced by IFN-γ epigenetic remodeling in response to anti–PD-1 therapy. J. Clin. Investig..

[32] Pozniak J., Pedri D., Landeloos E., Van Herck Y., Antoranz A., Vanwynsberghe L., Nowosad A., Roda N., Makhzami S., Bervoets G. (2024). A TCF4-dependent gene regulatory network confers resistance to immunotherapy in melanoma. Cell.

[33] Watt F.M., Frye M., Benitah S.A. (2008). MYC in mammalian epidermis: how can an oncogene stimulate differentiation?. Nat. Rev. Cancer.

[34] Jensen K.B., Watt F.M. (2006). Single-cell expression profiling of human epidermal stem and transit-amplifying cells: Lrig1 is a regulator of stem cell quiescence. Proc. Natl. Acad. Sci. USA.

[35] Gandarillas A., Watt F.M. (1997). c-Myc promotes differentiation of human epidermal stem cells. Genes Dev..

[36] San Juan L., Cagigal M.L., Fernandez-Flores A., Mayorga M., Gandarillas A. (2022). Protooncogene MYC drives human melanocyte melanogenesis and senescence. Cancer Gene Ther..

[37] Zinin N., Adameyko I., Wilhelm M., Fritz N., Uhlén P., Ernfors P., Henriksson M.A. (2014). MYC proteins promote neuronal differentiation by controlling the mode of progenitor cell division. EMBO Rep..

[38] Tsoi J., Robert L., Paraiso K., Galvan C., Sheu K.M., Lay J., Wong D.J.L., Atefi M., Shirazi R., Wang X. (2018). Multi-stage Differentiation Defines Melanoma Subtypes with Differential Vulnerability to Drug-Induced Iron-Dependent Oxidative Stress. Cancer Cell.

[39] Tirosh I., Izar B., Prakadan S.M., Wadsworth M.H., Treacy D., Trombetta J.J., Rotem A., Rodman C., Lian C., Murphy G. (2016). Dissecting the multicellular ecosystem of metastatic melanoma by single-cell RNA-seq. Science.

[40] Benci J.L., Xu B., Qiu Y., Wu T.J., Dada H., Twyman-Saint Victor C., Cucolo L., Lee D.S.M., Pauken K.E., Huang A.C. (2016). Tumor Interferon Signaling Regulates a Multigenic Resistance Program to Immune Checkpoint Blockade. Cell.

[41] Arifin M.Z., Leitner J., Egan D., Waidhofer-Soellner P., Kolch W., Zhernovkov V., Steinberger P. (2024). BTLA and PD-1 signals attenuate TCR-mediated transcriptomic changes. iScience.

[42] Benci J.L., Johnson L.R., Choa R., Xu Y., Qiu J., Zhou Z., Xu B., Ye D., Nathanson K.L., June C.H. (2019). Opposing Functions of Interferon Coordinate Adaptive and Innate Immune Responses to Cancer Immune Checkpoint Blockade. Cell.

[43] Bakhoum S.F., Ngo B., Laughney A.M., Cavallo J.A., Murphy C.J., Ly P., Shah P., Sriram R.K., Watkins T.B.K., Taunk N.K. (2018). Chromosomal instability drives metastasis through a cytosolic DNA response. Nature.

[44] Qiu J., Ye D., Chen X.E., Dangle N., Yoshor B., Zhang T., Shao Y., Nallamala V.C., Wang S., Ren D. (2024). Targeting Interferon-Driven Inflammatory Memory Prevents Epigenetic Evolution of Cancer Immunotherapy Resistance. bioRxiv.

[45] Srour N., Villarreal O.D., Hardikar S., Yu Z., Preston S., Miller W.H., Szewczyk M.M., Barsyte-Lovejoy D., Xu H., Chen T. (2022). PRMT7 ablation stimulates anti-tumor immunity and sensitizes melanoma to immune checkpoint blockade. Cell Rep..

[46] Chiappinelli K.B., Strissel P.L., Desrichard A., Li H., Henke C., Akman B., Hein A., Rote N.S., Cope L.M., Snyder A. (2015). Inhibiting DNA Methylation Causes an Interferon Response in Cancer via dsRNA Including Endogenous Retroviruses. Cell.

[47] Patel S.P., Othus M., Chen Y., Wright G.P., Yost K.J., Hyngstrom J.R., Hu-Lieskovan S., Lao C.D., Fecher L.A., Truong T.G. (2023). Neoadjuvant–Adjuvant or Adjuvant-Only Pembrolizumab in Advanced Melanoma. N. Engl. J. Med..

[48] Blank C.U., Lucas M.W., Scolyer R.A., Van De Wiel B.A., Menzies A.M., Lopez-Yurda M., Hoeijmakers L.L., Saw R.P., Lijnsvelt J.M. (2024). Neoadjuvant Nivolumab and Ipilimumab in Resectable Stage III Melanoma. N. Engl. J. Med..

[49] Chen Y., Samaraweera P., Sun T.-T., Kreibich G., Orlow S.J. (2002). Rab27b Association with Melanosomes: Dominant Negative Mutants Disrupt Melanosomal Movement. J. Invest. Dermatol..

[50] Chi A., Valencia J.C., Hu Z.Z., Watabe H., Yamaguchi H., Mangini N.J., Huang H., Canfield V.A., Cheng K.C., Yang F. (2006). Proteomic and Bioinformatic Characterization of the Biogenesis and Function of Melanosomes. J. Proteome Res..

[51] Snyder A., Makarov V., Merghoub T., Yuan J., Zaretsky J.M., Desrichard A., Walsh L.A., Postow M.A., Wong P., Ho T.S. (2014). Genetic Basis for Clinical Response to CTLA-4 Blockade in Melanoma. N. Engl. J. Med..

[52] Muquith M., Espinoza M., Elliott A., Xiu J., Seeber A., El-Deiry W., Antonarakis E.S., Graff S.L., Hall M.J., Borghaei H. (2024). Tissue-specific thresholds of mutation burden associated with anti-PD-1/L1 therapy benefit and prognosis in microsatellite-stable cancers. Nat. Cancer.

[53] Akbani R., Akdemir K., Aksoy B., Albert M., Ally A., Amin S., Arachchi H., Arora A., Auman J., Ayala B. (2015). Genomic Classification of Cutaneous Melanoma. Cell.

[54] McGranahan N., Furness A.J.S., Rosenthal R., Ramskov S., Lyngaa R., Saini S.K., Jamal-Hanjani M., Wilson G.A., Birkbak N.J., Hiley C.T. (2016). Clonal neoantigens elicit T cell immunoreactivity and sensitivity to immune checkpoint blockade. Science.

[55] Wang M.X., Mauch B.E., Williams A.F., Barazande-Pour T., Hoffmann F.A., Harris S.H., Lathrop C.P., Turkal C.E., Yung B.S., Paw M.H. (2025). Antigenic cancer persister cells survive direct T cell attack. bioRxiv.

[56] Larkin J., Chiarion-Sileni V., Gonzalez R., Grob J.J., Rutkowski P., Lao C.D., Cowey C.L., Schadendorf D., Wagstaff J., Dummer R. (2019). Five-Year Survival with Combined Nivolumab and Ipilimumab in Advanced Melanoma. N. Engl. J. Med..

[57] Egan D., Kreileder M., Nabhan M., Iglesias-Martinez L.F., Dovedi S.J., Valge-Archer V., Grover A., Wilkinson R.W., Slidel T., Bendtsen C. (2023). Small Gene Networks Delineate Immune Cell States and Characterize Immunotherapy Response in Melanoma. Cancer Immunol. Res..

[58] Grasso C.S. (2020). Conserved Interferon-γ Signaling Drives Clinical Response to Immune Checkpoint Blockade Therapy in Melanoma. Cancer Cell.

[59] Han J., Zhao Y., Shirai K., Molodtsov A., Kolling F.W., Fisher J.L., Zhang P., Yan S., Searles T.G., Bader J.M. (2021). Resident and circulating memory T cells persist for years in melanoma patients with durable responses to immunotherapy. Nat. Cancer.

[60] Zhang L., Wei X., Wang Z., Liu P., Hou Y., Xu Y., Su H., Koci M.D., Yin H., Zhang C. (2023). NF-κB activation enhances STING signaling by altering microtubule-mediated STING trafficking. Cell Rep..

[61] Lauss M., Phung B., Borch T.H., Harbst K., Kaminska K., Ebbesson A., Hedenfalk I., Yuan J., Nielsen K., Ingvar C. (2024). Molecular patterns of resistance to immune checkpoint blockade in melanoma. Nat. Commun..

[62] Priestley P., Baber J., Lolkema M.P., Steeghs N., de Bruijn E., Shale C., Duyvesteyn K., Haidari S., van Hoeck A., Onstenk W. (2019). Pan-cancer whole-genome analyses of metastatic solid tumours. Nature.

[63] Ewels P.A., Peltzer A., Fillinger S., Patel H., Alneberg J., Wilm A., Garcia M.U., Di Tommaso P., Nahnsen S. (2020). The nf-core framework for community-curated bioinformatics pipelines. Nat. Biotechnol..

[64] Love M.I., Huber W., Anders S. (2014). Moderated estimation of fold change and dispersion for RNA-seq data with DESeq2. Genome Biol..

[65] Danecek P., Bonfield J.K., Liddle J., Marshall J., Ohan V., Pollard M.O., Whitwham A., Keane T., McCarthy S.A., Davies R.M., Li H. (2021). Twelve years of SAMtools and BCFtools. GigaScience.

[66] Butler A., Hoffman P., Smibert P., Papalexi E., Satija R. (2018). Integrating single-cell transcriptomic data across different conditions, technologies, and species. Nat. Biotechnol..

[67] Trapnell C., Cacchiarelli D., Grimsby J., Pokharel P., Li S., Morse M., Lennon N.J., Livak K.J., Mikkelsen T.S., Rinn J.L. (2014). The dynamics and regulators of cell fate decisions are revealed by pseudotemporal ordering of single cells. Nat. Biotechnol..

[68] Aibar S., González-Blas C.B., Moerman T., Huynh-Thu V.A., Imrichova H., Hulselmans G., Rambow F., Marine J.C., Geurts P., Aerts J. (2017). SCENIC: single-cell regulatory network inference and clustering. Nat. Methods.

[69] Andreatta M., Carmona S.J. (2021). Robust and scalable single-cell gene signature scoring. Comput. Struct. Biotechnol. J..

[70] Renevey A. (2024). nf-core/rnafusion: 3.0.2.

[71] Dobin A., Davis C.A., Schlesinger F., Drenkow J., Zaleski C., Jha S., Batut P., Chaisson M., Gingeras T.R. (2013). STAR: ultrafast universal RNA-seq aligner. Bioinformatics.

[72] Uhrig S., Ellermann J., Walther T., Burkhardt P., Fröhlich M., Hutter B., Toprak U.H., Neumann O., Stenzinger A., Scholl C. (2021). Accurate and efficient detection of gene fusions from RNA sequencing data. Genome Res..

[73] Bendall M.L., de Mulder M., Iñiguez L.P., Lecanda-Sánchez A., Pérez-Losada M., Ostrowski M.A., Jones R.B., Mulder L.C.F., Reyes-Terán G., Crandall K.A. (2019). Telescope: Characterization of the retrotranscriptome by accurate estimation of transposable element expression. PLoS Comput. Biol..

[74] Xie Z., Bailey A., Kuleshov M.V., Clarke D.J.B., Evangelista J.E., Jenkins S.L., Lachmann A., Wojciechowicz M.L., Kropiwnicki E., Jagodnik K.M. (2021). Gene Set Knowledge Discovery with Enrichr. Curr. Protoc..

[75] Song L., Cohen D., Ouyang Z., Cao Y., Hu X., Liu X.S. (2021). TRUST4: immune repertoire reconstruction from bulk and single-cell RNA-seq data. Nat. Methods.

[76] Wolf Y., Bartok O., Patkar S., Eli G.B., Cohen S., Litchfield K., Levy R., Jiménez-Sánchez A., Trabish S., Lee J.S. (2019). UVB-Induced Tumor Heterogeneity Diminishes Immune Response in Melanoma. Cell.

[77] Li H., Durbin R. (2009). Fast and accurate short read alignment with Burrows–Wheeler transform. Bioinformatics.

[78] Hanssen F., Garcia M.U., Folkersen L., Pedersen A.S., Lescai F., Jodoin S., Miller E., Seybold M., Wacker O., Smith N. (2024). Scalable and efficient DNA sequencing analysis on different compute infrastructures aiding variant discovery. NAR Genom. Bioinf..

[79] Benjamin D., Sato T., Cibulskis K., Getz G., Stewart C., Lichtenstein L. (2019). Calling Somatic SNVs and Indels with Mutect2. bioRxiv.

[80] Nik-Zainal S., Van Loo P., Wedge D.C., Alexandrov L.B., Greenman C.D., Lau K.W., Raine K., Jones D., Marshall J., Ramakrishna M. (2012). The Life History of 21 Breast Cancers. Cell.

[81] Gillis S., Roth A. (2020). PyClone-VI: scalable inference of clonal population structures using whole genome data. BMC Bioinf..

