# Supplementary material for: Chronic interferon signaling at baseline is associated with acquired resistance to immunotherapy in metastatic melanoma
Source: Cell Rep Med. 2026 Aug 7;7(8):102981. doi: 10.1016/j.xcrm.2026.102981 (PMC13522794; doi:10.1016/j.xcrm.2026.102981)
Supplement: Supplementary file 1 [file mmc1.pdf]

Cell Reports Medicine, Volume *7*

Supplemental information

Chronic interferon signaling at baseline is associated with acquired resistance to immunotherapy in metastatic melanoma

Donagh Egan, Ben Shum, Rabia Fidan, Walter Kolch, Donal Brennan, Irene Lobon, and Samra Turajlic

Supplementary Figures

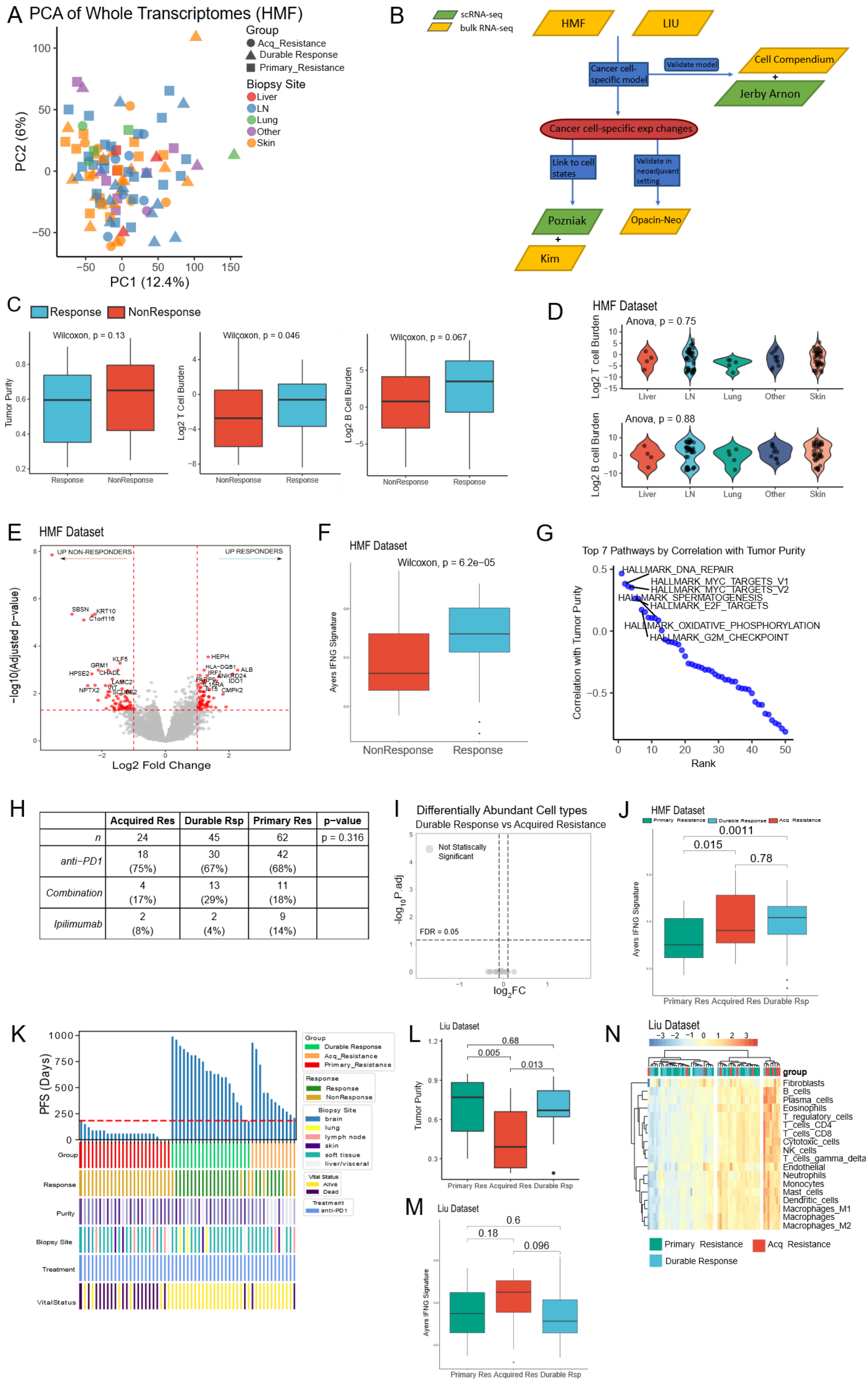

	Acquired Res	Durable Rsp	Primary Res	p–value
n	24	45	62	p = 0.316
anti–PD1	18 (75%)	30 (67%)	42 (68%)	
Combination	4 (17%)	13 (29%)	11 (18%)	
Ipilimumab	2 (8%)	2 (4%)	9 (14%)	

Supplementary Figure 1. Clinical and molecular features associated with initial and durable response in the Liu and HMF datasets. A) Principal component analysis of gene expression profiles from HMF patients, colored by sample site. B) The datasets included in the study and their respective analytical purposes. C) Tumor purity (left), T cell burden (middle), and B cell burden (right) in responders and non-responders. T and B cell burden values represent the relative abundance of clonally rearranged T and B cell transcripts in a tumor. D) T cell burden (top) and B cell burden (bottom) across sample sites. T and B cell burden values represent the relative abundance of clonally rearranged T and B cell transcripts. E) The DEGs between responders and non-responders. The genes delineated in red satisfied the significance (FDR < 0.05) and fold change (log2FC > 1) thresholds. F) The activity of the IFNG-related signature published by Ayers et al[4] in responders and non-responders. G) Pathways from the Hallmarks database, ranked according to their correlation with tumor purity in HMF dataset. H) A Chi-sq distribution table of patients in the duration of response groups who received anti-PD-1, anti-CTLA-4, or combination therapy (defined as anti-PD-1 plus anti-CTLA-4). To improve statistical power, patients without bulk RNA-seq data were also included (n = 131). I) DR (durable response) and AR (acquired resistance) comparison of ConsensusTME inferred cell types in the HMF dataset. A two-sided Wilcoxon rank-sum test with BH correction was used. J) The activity of the established IFNG-related signature published by Ayers et al[4] in the duration of response groups. K) Annotation plot showing clinical information and response outcomes for the Liu dataset. Each column represents a patient. Patients are ordered by duration of response group, and within each group by duration of response follow up. The horizontal red line denotes the minimum follow-up required for assignment to a duration of response group. L) Tumor purity estimates for the duration of response groups in the Liu et al dataset. M) The activity of the established IFNG-related signature[4] in the duration of response groups from the Liu dataset, respectively. N) Hierarchical clustering of the Liu et al dataset according to deconvolution derived estimates of cell type abundances, annotated according to duration of response group.

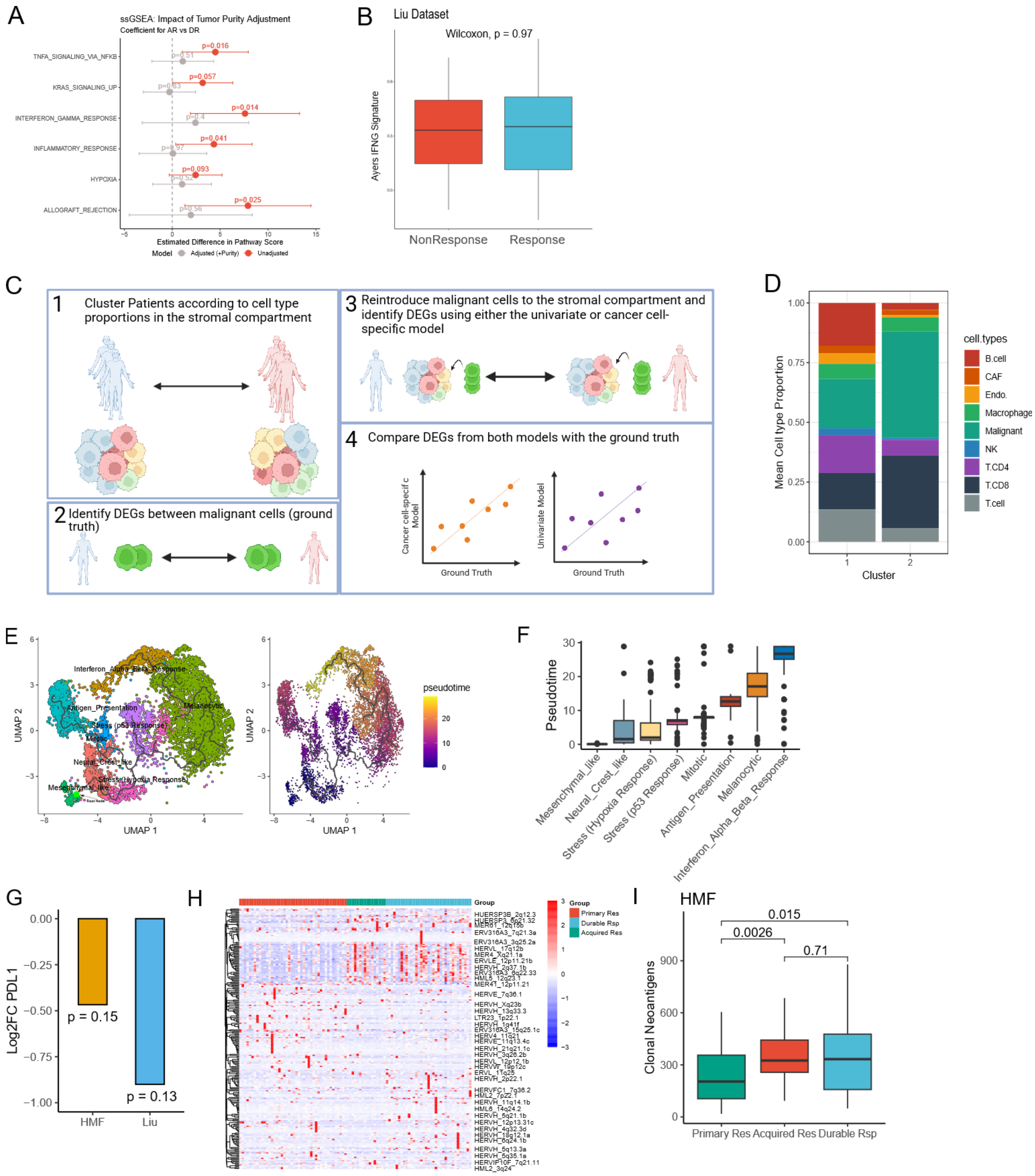

Supplementary Figure 2. The cancer cell-dependent model and validations in external datasets. A) The estimated difference in pathway activity between DR and AR patients, calculated using a standard univariate model, or a tumor purity-adjusted model. Pathway activity was inferred using ssGSEA. B) The activity of the established IFNG-related signature[4] in the response groups from the Liu dataset, respectively. C) The workflow used to validate the cancer cell-dependent model in the Jerby-Arnon scRNA-seq dataset.[22] D) The proportion of each cell type in the stromal compartment of the tumor clusters that were compared to identify a ground truth for differences in malignant cell expression. E) UMAP of melanoma cells from Pozniak et al.,[38] overlaid with a Monocle3-inferred trajectory, tracing differentiation from mesenchymal stem cells (left panel) and corresponding pseudotime scores (right panel). F) The pseudotime scores for each melanoma cell phenotype. G) The difference in *PDL1* expression between DR (reference group) and AR patients using the cancer cell-dependent model. H)

Differentially expressed HERVs (FDR < 0.05; likelihood ratio test) across the duration of response groups. I) The number of clonal neoantigens in the duration of response groups, compared using a Wilcoxon rank-sum test (two-sided).

Supplementary Tables

Supplementary Table S1. HMF Patient Characteristics (w/RNA-seq) and Distribution of Metastases Across Biopsy Sites

**Patient Characteristic (w/RNA-seq)**	**All (n=108)**
**Sex (N, %)**	
Male	64 (59)
Female	44 (41)
Age (median, range)	66 (26–94)
**Melanoma subtype (N, %)**	
Cutaneous	108 (100)
**Stage (N, %)**	
M1a (LN only)	17 (16%)
M1b (Lung but not liver/bone/brain)	21 (19%)
M1c (liver/bone but not brain)	18 (17%)
M1d (brain)	12 (11%)
NA	40 (37%)
**Treatment (N, %)**	
Combination Ipilimumab Nivolumab	25 (23)
PD1 monotherapy	76 (70)
Ipilimumab	7 (7)
**Treatment outcome (N, %)**	
Primary resistance	44 (44)
Acquired resistance	16 (15)
Durable response	36 (33)
Not Assigned	12 (11)

Biopsy Site	Mts Brain	Mts Liver	Mts Lung	Mts Bone	Mts LN
Subcutaneous tissue	6	9	19	8	27
LN	4	6	10	6	27
Lung	1	1	1	0	0
Bone	0	0	0	0	0
Cutaneous	1	0	1	0	1
Liver	0	0	0	0	0

Supplementary Table S5. Liu et al Patient Characteristics

Patient Characteristic	All (n=57)
**Sex (N, %)**	
Male	35 (61)
Female	22 (39)
**Melanoma subtype (N, %)**	
Cutaneous	42 (74)
Unknown Primary	9 (16)
Acral	3 (5)
Mucosal	3 (5)
**Stage (N, %)**	
M1a	3 (5)
M1b	10 (18)
M1c	34 (60)
Unresectable stage 3	10 (18)
**Treatment (N, %)**	
PD1 monotherapy	34 (100)
**Treatment outcome (N, %)**	
Primary resistance	24 (42)

Acquired resistance	12 (21)
Durable response	21 (37)

Supplementary Table S12. OpACIN-neo Patient Characteristics

Patient Characteristic	All (n=63)
**Sex (N, %)**	
Male	37 (59)
Female	26 (41)
**Melanoma subtype (N, %)**	
Cutaneous	43 (68)
Unknown Primary	20 (32)
**Stage (N, %)**	
Stage III	63 (100)
**Treatment (N, %)**	
Neoadjuvant Ipilimumab plus Nivolumab	63 (100)
**Treatment outcome (N, %)**	
Pathological Response	46 (73)
No Pathological Response	17 (27)
